# An acidic pH independent piperazine–TPE AIEgen as a unique bioprobe for lysosome tracing†
†Electronic supplementary information (ESI) available: NMR, single crystal X-ray crystallography of PIP–TPE, live cell and fixed cell fluorescence imaging, MTT, photostability, and theoretical calculations. CCDC 1555412. For ESI and crystallographic data in CIF or other electronic format see DOI: 10.1039/c7sc03515b


**DOI:** 10.1039/c7sc03515b

**Published:** 2017-09-18

**Authors:** Yuanjing Cai, Chen Gui, Kerim Samedov, Huifang Su, Xinggui Gu, Shiwu Li, Wenwen Luo, Herman H. Y. Sung, Jacky W. Y. Lam, Ryan T. K. Kwok, Ian D. Williams, Anjun Qin, Ben Zhong Tang

**Affiliations:** a Guangdong Innovative Research Team , State Key Laboratory of Luminescent Materials and Devices , South China University of Technology , Guangzhou 510640 , China . Email: msqinaj@scut.edu.cn; b Department of Chemistry , Hong Kong Branch of Chinese National Engineering Research Center for Tissue Restoration and Reconstruction , The Hong Kong University of Science & Technology , Clear Water Bay , Kowloon , Hong Kong , China . Email: tangbenz@ust.hk; c Department of Chemistry , University of British Columbia , 2036 Main Mall , Vancouver , British Columbia , Canada V6T 1Z1

## Abstract

Lysosomes are involved in a multitude of cellular processes and their dysfunction is associated with various diseases. They are the most acidic organelles (pH 3.8–6.6, size 0.1–1.2 μm) with the highest viscosity (47–190 cP at 25 °C) in the cell. Because of their acidity, pH dependent non-AIE active fluorescent lysosomal probes have been developed that rely on protonation inhibited photoinduced electron transfer (PET). In this work, an acidic pH independent lysosome targetable piperazine–TPE (PIP–TPE) AIEgen has been designed with unique photophysical properties making it a suitable probe for quantifying viscosity. In a non-aggregated state PIP–TPE shows deep-blue emission as opposed to its yellowish-green emission in the bulk. It possesses high specificity for lysosomes with negligible cytotoxicity and good tracing ability due to its better photostability compared to LysoTracker Red. In contrast to most known lysosome probes that rely solely on PET, restriction of intramolecular motion (RIM) due to the larger viscosity inside the lysosomes is the mechanism responsible for PIP–TPE’s fluorescence. PIP–TPE’s high selectivity is attributed to its unique molecular design that features piperazine fragments providing a perfect balance between lipophilicity and polarity.

## Abstract 2

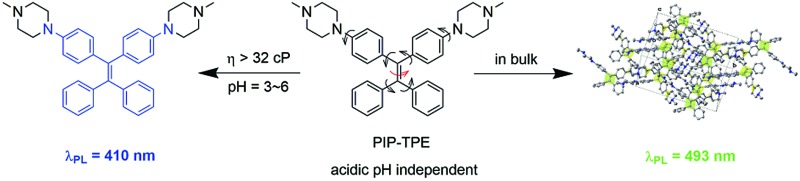
PIP–TPE’s fluorescence turns on blue due to the large viscosity of lysosomes which restricts intramolecular motions but it red-shifts in the bulk.

## Introduction

Lysosomes, organelles in most eukaryotic cells, play an important role in maintaining cellular homeostasis, including recycling damaged organelles, digesting macromolecules, as well as participating in intracellular signaling and plasma membrane repair *etc*.^1^–^3^ It has been reported that the dysfunction of lysosomes is associated with many diseases, such as lysosomal storage diseases, neurodegenerative diseases, cardiovascular diseases, inflammation and even cancer.^4^–^9^ Hence, more and more research on lysosome selectively-targeting probes and lysosomal sensors has been done to better understand the status of lysosomes.^10^–^14^ Up to now, formaldehyde, nitroreductase, intracellular thiols, viscosity and many specific analytes in lysosomes have been well recognized.^1^,^12^,^14^–^16^

With proton-pumping vacuolar ATPases in lysosomes or stimulation caused by a lysosomotropic agent that can inhibit autophagy and protein degradation by raising the lysosomal pH, lysosomes can maintain their luminal environment under acidic conditions within pH 3.8–6.6.^3^,^17^,^18^ Thus, lysosomes are the most acidic organelles compared to other subcellular components (pH *ca.* 7.4).^19^ Based on this feature, most pH responsive lysosomal probes with amino groups have been designed to target the acidic environment within lysosomes,^19^–^22^ the representatives of which are LysoTracker probes.^10^,^13^,^23^ Except for commercial lysosomal probes, morpholine and piperazine units are widely employed as lysosome specific targeting functional groups.^1^,^12^–^14^,^17^,^18^,^24^–^29^ To guarantee lysosome selectivity, the functional groups, hydrophilicity as well as the polarity of the targeting probe play important roles. Since the dipole moment of piperazine is similar to that of morpholine,^30^ and one more N atom in piperazine endows it with a better ability to make hydrogen bonds, piperazine is more hydrophilic than morpholine. The two piperazine-functionalized tetraphenylethylene (TPE) has good lysosome selectivity (*vide infra*), while the two morpholine-functionalized TPE cannot selectively light up the lysosome but stains the entire hydrophobic region.^31^ This might be due to the hydrophobic interactions of TPE with other cellular organelles competing with the driving force from the morpholine, and thus reducing the lysosome selectivity.

The fluorescence of LysoTracker Red, LysoTracker Green and most pH responsive lysosomal probes is turned on due to the protonation of the N atom which effectively eliminates photoinduced electron transfer (PET), the phenomenon that is responsible for fluorescence quenching.^11^,^17^,^18^,^21^,^24^,^29^ Thus, the lysosomal probes with the working mechanism of protonation prohibiting PET are highly reliant on the pH value and their emission intensity is unstable and varies significantly with a varying acidity of the microenvironment.^17^,^21^,^22^,^29^ On the other hand, a high background luminescent noise signal can be observed due to insufficient fluorescence quenching through PET outside of the lysosome, and some LysoTracker probes in such a case^22^,^31^ do not allow a high signal to noise ratio to be obtained.

Additionally, the commercial LysoTracker probes have the aggregation-caused quenching (ACQ) effect. Thus, the working concentration of LysoTracker Red is usually low. Furthermore, it is not photostable due to its BODIPY-based structure, which makes it unsuitable for long-term lysosome trace tracing. Moreover, the LysoTracker probes also have small Stokes shifts (less than 20 nm) which is highly disadvantageous for bioimaging applications. On the contrary, the development of AIEgens (aggregation-induced emission luminogens) allows a higher working concentration to be used with good photostability.^31^,^32^ However, there are only a few AIEgens developed for targeting lysosomes and for most AIE lysosomal probes developed up till now, their photoluminescence (PL) changes within the lysosomal pH range, which results in low localizing stability against pH changes.^26^,^31^ Although the fluorescence emission of most lysosomal probes is pH-dependent to a varying degree, and some of them do allow for monitoring lysosomal pH changes,^12^,^17^,^18^,^27^,^29^ there are few studies that have focused on the development of intralysosomal pH stable fluorescent AIE probes. However, a pH-independent lysosomal probe that would allow monitoring of other fundamental microenvironment parameters, such as the viscosity (67–170 ± 20 cP at 25 °C) of the lysosome, would be of great use for sensing microviscosity which can reflect the status, integrity and function of this organelle.^11^,^33^

**Fig. 1:**
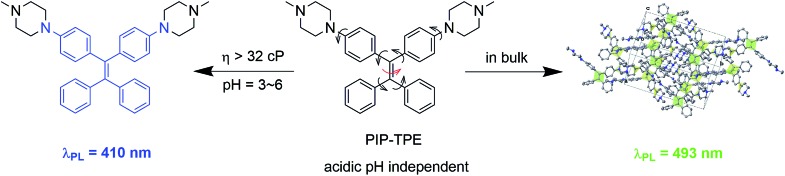
PIP–TPE’s fluorescence turns on blue due to the higher viscosity that restricts intramolecular motion (RIM) in lysosomes but red-shifts in the bulk (*λ*_ex_ = 360 nm).

In this work, we present PIP–TPE (Fig. 1), an acidic pH-stable lysosomal tracing AIEgen with viscosity sensitivity achieved by attaching the hydrophilic piperazine unit to the fluorescent tetraphenylethylene (TPE). In contrast to the working mechanism of PET for most lysosome probes, the PIP–TPE’s turn-on fluorescence in lysosomes can be attributed to the viscosity restricting intramolecular rotation and C=C twisting (Fig. 1). The fluorescence emission maximum of PIP–TPE shifts from deep blue in its non-aggregated state to yellowish-green in the bulk. Additionally, its photophysical properties are not influenced by protonation. Compared to LysoTracker probes and known lysosomal AIE probes, PIP–TPE shows good photostability and a high signal-to-noise ratio, and its fluorescence is not affected by the acidic pH variation in the microenvironment.

## Results and discussion

### Synthesis and characterization of PIP–TPE

**Scheme 1:**
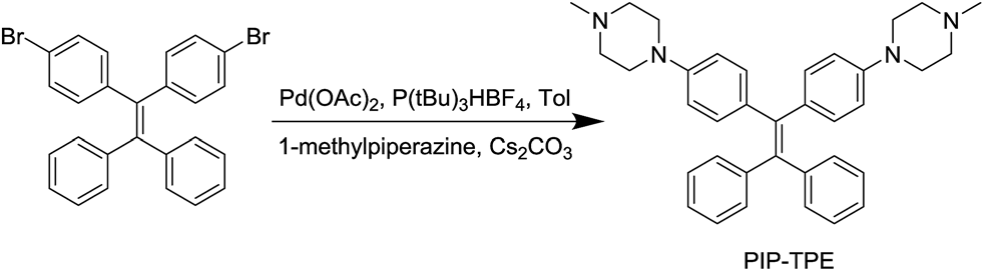
Synthetic route to PIP–TPE.

**Fig. 2:**
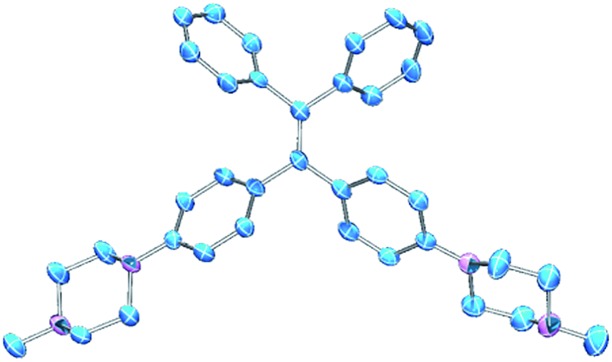
ORTEP drawing of PIP–TPE (CCDC: 1555412). All thermal ellipsoids are shown at 50% thermal probability. All H atoms are omitted for clarity.

PIP–TPE was synthesized according to the synthetic route shown in Scheme 1. The isolated compound was characterized by ^1^H-NMR, ^13^C-NMR and high resolution mass spectrometry. The structure was determined by single-crystal X-ray diffraction (see ESI† for details). The crystal structure of PIP–TPE is shown in Fig. 2, which confirms its correct structure.

### The photophysical properties of PIP–TPE

**Fig. 3:**
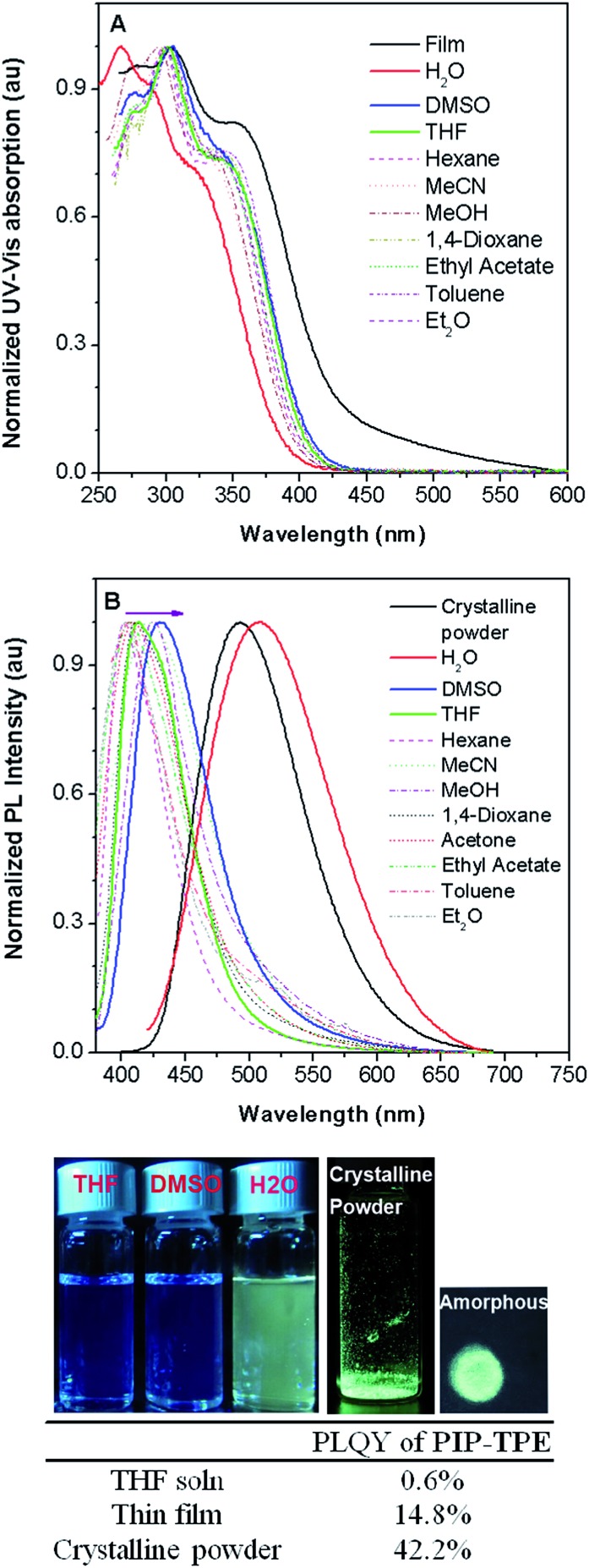
(A) Normalized UV absorption of PIP–TPE in different solvents (0.8 × 10^–5^ M) and as a thin film. (B) Normalized PL spectra of PIP–TPE in different solvents (0.8 × 10^–5^ M) and as a crystalline powder (*λ*_ex_ = 360 nm). Photographs of PIP–TPE in different solvents, as an amorphous powder obtained from an evaporated DMSO solution on filter paper and as a crystalline powder taken under 365 nm UV irradiation using a hand-held UV lamp. Inserted table: the absolute photoluminescence quantum yields (PLQYs) of PIP–TPE in THF solution, as a thin film and as a crystalline powder (*λ*_ex_ = 303 nm).

The photophysical properties of PIP–TPE were studied in solvents of different polarity and in the solid state. As shown in Fig. 3A, the PIP–TPE samples dissolved in organic solvents have similar UV-Vis absorptions with peaks at around 275 nm, 302 nm, and 346 nm and absorption bands at 250–450 nm. While its absorption peaks in a thin film are observed at around 277 nm, 305 nm and 356 nm with marked band broadening to 600 nm, its absorption as an amorphous suspension in neutral water (Fig. S4†) is blue shifted with peaks at 267 nm, 290 nm and 325 nm. This might be caused by hydrogen bonding between the water molecules and nitrogen atoms of the piperazine moiety in PIP–TPE that is known to allow the formation of hydrogen-bonded water-containing PIP–TPE aggregates.^34^

In PIP–TPE, the nitrogen atoms of aniline can be considered as donors, while the TPE unit can be treated as an acceptor (see HOMO–LUMO in Fig. S12†). The orbital distribution indicates that PIP–TPE has a very weak D–A structure. Thus, its solvatochromic effect in solvents of different polarity is rather subtle,^35^*e.g.*, its PL emission maxima in organic solvents vary from 404 nm in hexane to 413 nm in THF and 430 nm in DMSO (Fig. 3B). However, as PIP–TPE does not dissolve well in neutral H_2_O and tends to form micro-sized aggregates (particle size beyond the nanoscale is further referred to as the bulk) (Fig. S4†), its PL emission in such amorphous aggregates has a large red shift with *λ*_em_ = 509 nm (Fig. 3B). Similarly, PIP–TPE as a crystalline powder has an emission peak at 493 nm. The absolute quantum yield of PIP–TPE in THF solution is 0.6%, while as a thin film and crystalline powder its efficiencies are 14.8% and 42.2%, respectively. Additionally, the absolute fluorescence quantum yields of PIP–TPE (10^–5^ M) in MeOH with different fractions of glycerol increase as the content of glycerol increases (Fig. S5†). Thus, the compound PIP–TPE is AIE-active.

### The pH and viscosity sensitivity of PIP–TPE

**Fig. 4:**
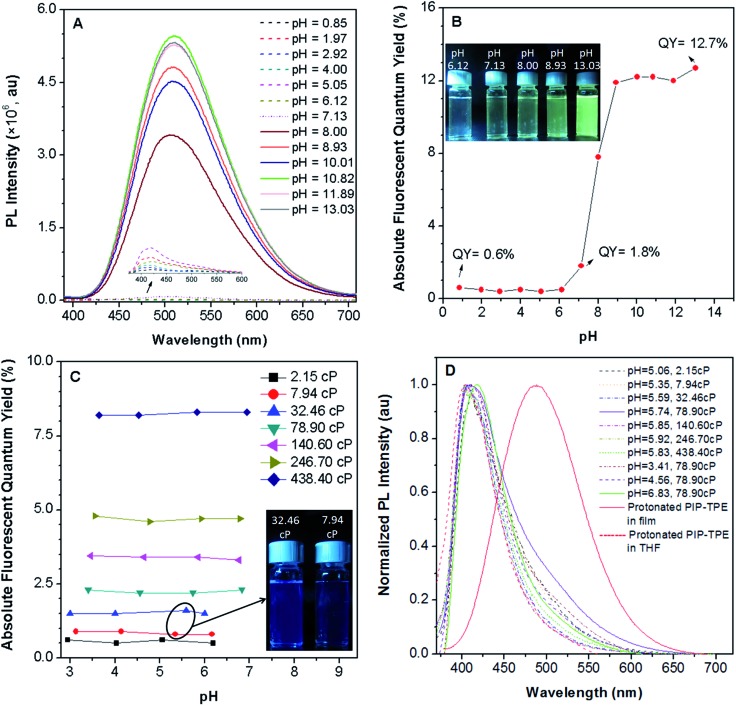
(A) Photoluminescence (PL) intensity and (B) absolute fluorescence quantum yields of PIP–TPE in pH aqueous buffer solutions (*λ*_ex_ = 360 nm), inset: photographs of PIP–TPE in pH buffer solutions taken under illumination of a UV lamp at 365 nm; (C) the absolute fluorescence quantum yields of PIP–TPE in different acidic buffer solutions at various viscosities (*λ*_ex_ = 360 nm), inset: photographs of PIP–TPE in pH 5.59 solution at a viscosity of 32.46 cP and in pH 5.35 solution at a viscosity of 7.94 cP taken under illumination of a UV lamp at 365 nm; (D) the normalized PL intensities of PIP–TPE in acidic buffer solutions of different viscosities (*λ*_ex_ = 360 nm), and protonated PIP–TPE in a thin film (*λ*_ex_ = 360 nm) and in THF (*λ*_ex_ = 300 nm). All of the data for solutions in 0.8 × 10^–5^ M were collected at 25 °C.

PIP–TPE’s fluorescence intensity (*λ*_em_ = 420 nm) is not sensitive to pHs below 7 (Fig. 4A) as the fluorescence quantum yields remain almost identical (Fig. 4B). Additionally, the fluorescence of PIP–TPE emitted in the acidic aqueous buffer solutions is weak and its fluorescence quantum yields are less than 1%. However, we note that PIP–TPE’s fluorescence obviously changes (*λ*_em_ = 509 nm) and turns on when its fluorescence quantum yield is above 1.8% in the aqueous buffer solution of pH 7.13, and the quantum yield maximum value reaches 12.7% with a large extent of increased fluorescence intensity in the basic aqueous solution of pH 13.03 (Fig. 4B). In fact, the protonated PIP–TPE should have a lower propensity to form large aggregates in the acidic milieu. The particle sizes of PIP–TPE in aqueous buffer solutions of pH 0.85, 5.05 and 13.03 (Fig. S6†) are 89 nm, 385 nm and 24.2 μm, respectively. This reveals that the protonated PIP–TPE molecules are unlikely to form large (micro-scale) aggregates in the aqueous solutions of acidic pH values. Thus, the low fluorescence quantum yield of PIP–TPE in acidic milieu with aqueous viscosity can be attributed to better solubility and a diminished proclivity to aggregate due to the protonation of PIP–TPE resulting in intermolecular repulsion. The higher fluorescence quantum yield of PIP–TPE in basic aqueous solution is due to the larger aggregation induced emission effect.

In an intracellular system lysosomes are the most acidic organelles with the highest viscosity,^11^,^33^ therefore the fluorescence properties of PIP–TPE in a series of viscosity gradients of acidic aqueous buffer solutions and glycerol in various proportions to simulate the intralysosomal milieu were also studied. Along with the increased viscosity from 2.15 cP to 438.40 cP, PIP–TPE’s fluorescence quantum yields increase remarkably from 0.6% to 8.2% and remain almost the same at a specific viscosity without any significant dependence on the pH from 2.95 to 6.94 (Fig. 4C). For example, the fluorescence quantum yields of PIP–TPE in the acidic solutions with viscosities of 7.94 cP are all *ca.* ∼0.9%. However, the fluorescence of its acidic solutions at viscosities of 32.46 cP turns on obviously with fluorescence quantum yields of ∼1.5% (Fig. 4C). Moreover, the fluorescence quantum yields of PIP–TPE are ∼2.3% in acidic solutions with viscosities of 78.90 cP, ∼3.4% at 140.60 cP and ∼4.7% at 246.70 cP. On the other hand, the PL emission peak of PIP–TPE in solutions of similar pH but different viscosities and different pH but the same viscosity is at around 410–419 nm. For example, PIP–TPE in pH 5–6 buffer solutions with viscosities ranging from 2.15 cP to 438.40 cP and in pH 3.41–6.83 buffer solutions with viscosities of 78.90 cP emit deep blue light (Fig. 4D). Furthermore, the emission peak of the protonated PIP–TPE as a thin film appears at 488 nm (Fig. 4D), which is similar to that of PIP–TPE as a crystalline powder (Fig. 3B), indicating that the PL emission of PIP–TPE is not influenced by protonation. The above discovery indicates that the protonation of PIP–TPE does not have any significant influence on its photophysical properties in the non-aggregated state or in the bulk (Fig. 3B and 4D), but does influence its scale of aggregation. PIP–TPE’s fluorescence is viscosity dependent but is rather independent of acidic pH changes. Thus, the quantum yield of PIP–TPE is suitable for quantifying viscosity.

### Biological applications of PIP–TPE

**Fig. 5:**
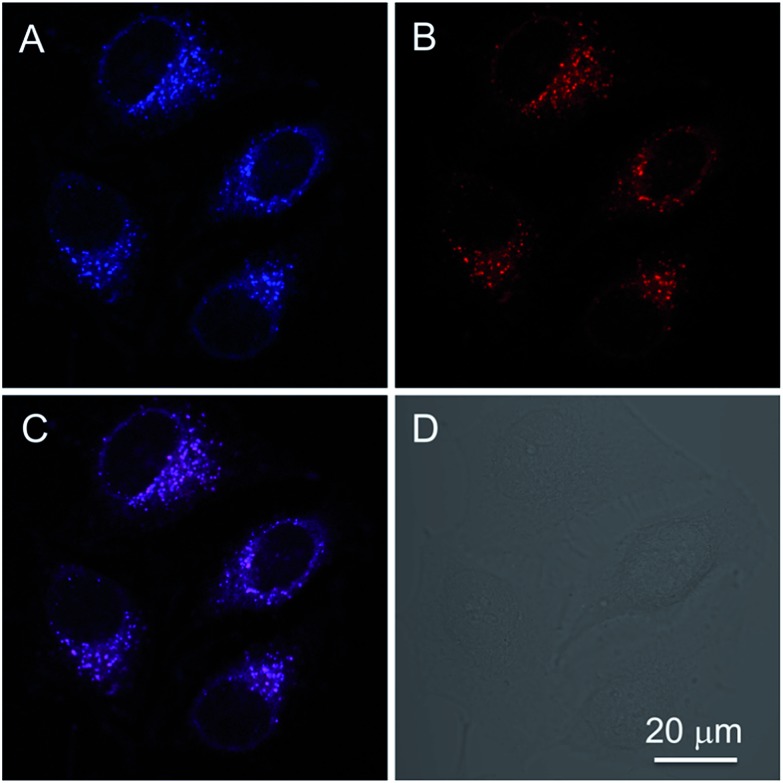
(A and B) Confocal images of HeLa cells co-stained with 1 μM PIP–TPE and 200 nM LysoTracker Red for 15 min, (C) merged image of (A) and (B) and (D) bright field; excitation wavelength: 405 nm for PIP–TPE; 561 nm for LysoTracker Red. Scale bar = 20 μm.

In addition to the photophysical properties of PIP–TPE outside of the cell, we also studied its fluorescence bioapplications *in vitro*. PIP–TPE shows good selectivity towards lysosomes and displays strong localized fluorescence within lysosomes. HeLa cells were co-stained with PIP–TPE and the commercial lysosome probe LysoTracker Red for 15 min at 37 °C. The PIP–TPE in HeLa cells showed blue fluorescence under a microscope (Fig. S8B†), while LysoTracker Red showed red fluorescence under an excitation wavelength of 561 nm (Fig. 5B). The merged image (Fig. 5C) indicates that the two images (Fig. 5A and B) overlap very well with a correlation coefficient of 0.82, confirming that PIP–TPE can specifically localize in the lysosomes of living cells. Although the TPE unit in PIP–TPE is hydrophobic, the incorporation of two hydrophilic piperazine units into TPE increases its solubility in aqueous acidic milieu, thus resulting in a good signal-to-noise ratio when it targets the lysosome (Fig. 5A).^31^

It is difficult to precisely replicate intralysosomal conditions (*e.g.*, polarity *etc*.) *in vitro*. Since protonated PIP–TPE molecules would naturally display a lower propensity to form large (micro-scale) aggregates in acidic media (*vide supra*), we assume that in lysosomes they will primarily exist in the form of individual protonated molecules and/or nano-scale aggregates with the upper size limit determined by the size of the lysosomes (100 nm to 1.2 μm).^36^ Thus, PIP–TPE’s fluorescence in lysosomes under a microscope is blue (Fig. S8B†). As its fluorescence is not affected by the pH variation in the acidic milieu but is highly sensitive to the viscosity, PIP–TPE barely fluoresces in acidic aqueous solutions of low viscosity (*e.g.*, outside lysosomes *vide supra*). However, PIP–TPE’s fluorescence does turn on in lysosomes due to its large viscosity.^11^,^33^ Furthermore, the comparison of fixed cell and live cell experiments (Fig. S8†) shows that once the HeLa cell is fixed, PIP–TPE loses its selectivity towards lysosomes and lights up in the whole cytosol. We speculate that PIP–TPE in live HeLa cells also diffuses in the cytosol without any noticeable effect due to cytosol’s low viscosity. Thus, the good selectivity of PIP–TPE towards lysosomes can be attributed to the piperazine functional groups in this molecule as well as the highly viscous intralysosomal milieu rather than a protonation effect (*e.g.* PET).

For a real-time tracking probe, low cell toxicity and good biocompatibility are key features for living cell imaging. Therefore, the cell viability with incubation of PIP–TPE was further investigated using an MTT (3-(4,5-dimethylthiazol-2-yl)-2,5-diphenyltetrazolium bromide) assay. The result (Fig. S9†) shows that for a higher concentration of 5 μM of PIP–TPE used for incubation than the optimized level for staining (1 μM), there is no obvious cytotoxicity to HeLa cells.

**Fig. 6:**
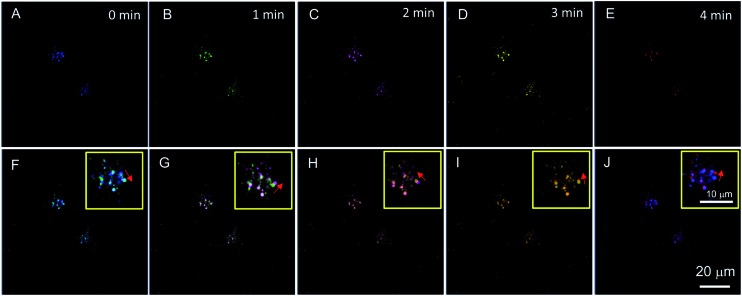
(A–E) Confocal images of HeLa cells incubated with 1 μM PIP–TPE for 15 min and then stimulated using 3 μM chloroquine for (A) 0 min, (B) 1 min, (C) 2 min, (D) 3 min, and (E) 4 min. Merged images at two different points in time: (F) 0 and 1 min, (G) 1 and 2 min, (H) 2 and 3 min, (I) 3 and 4 min, and (J) 0 and 4 min, in the yellow squares: enlarged image for a specific lysosome with a red arrow indicating its direction of movement. Excitation wavelength: 405 nm. Scale bar = 20 μm.

The commercial lysosome probe LysoTracker Red cannot be used to trace the migration of lysosomes due to its serious photobleaching after a second time of excitation.^23^ However, except for a specific lysosomal targeting ability, PIP–TPE has better photostability than LysoTracker Red (Fig. S10†) and shows the capability to trace lysosomes. Chloroquine, a typical lysosomal drug for driving lysosomal migration without inducing any other apparent disturbances in cells, was used to stimulate lysosomes moving in the HeLa cells. A time series of confocal microscopy images with the aid of PIP–TPE was recorded over a period of 4 min (Fig. 6A–E). By merging the images taken at different points in time, the drift direction and distance of a lysosome can be clearly observed (Fig. 6F–J). The short-term shifts can be unambiguously traced in the merged images taken at any two continuous times (Fig. 6F–I). It reveals that the locations of the lysosomes changed little by little with random drift directions. Through analyzing the merged images taken at 0 min and 4 min (Fig. 6J), an obvious large shift of the lysosome is observed. Such a good image quality of PIP–TPE for tracing lysosomes should be ascribed to the good photostability of PIP–TPE, bright fluorescence within the lysosomes and the negligible background fluorescence.

### Theoretical calculations of PIP–TPE and PIP–TPEH^+^

In an attempt to gain more insight and explore possible fluorescence mechanisms operative in PIP–TPE in lysosomes and in the bulk, DFT/TD-DFT calculations were performed using the B3LYP functional^37^–^40^ and the 6-31G(d) basis set as implemented in the D0.1 version of the Gaussian 09 software package.^41^ The bulk solvent effect (water) in these calculations was modeled using the polarizable continuum model (PCM)^42^ as implemented in the D0.1 version of the Gaussian 09 package. The photophysical properties of PIP–TPE in the bulk were analyzed using ONIOM (QM:MM),^43^–^46^ an integrated quantum mechanics:molecular mechanics (QM:MM) method. In the present work, a two-layer ONIOM QM:MM model was employed in which a PIP–TPE molecule was treated by DFT/TD-DFT, while the rest of the bulk was treated by a classical force field (UFF).^47^

Given the lysosomes’ acidic milieu, in lysosomes PIP–TPE will exist for the most part in its protonated form. Based on the p*K*_a_ values of the structurally very similar dimethylpiperazine (p*K*_a_ = 8.3) and diethylamine (p*K*_a_ = 6.6) in water reported elsewhere,^48^ it appears reasonable to assume that the peripheral tertiary nitrogen atom(s) of the piperazine moiety(ies) would be charge carriers in such a protonated species. Further experiments with PIP–TPE and concentrated aqueous HCl allowed the isolation and identification (NMR and MALDI-ToF) of the protonation product of PIP–TPE as its mono hydrochloride, PIP–TPE·HCl. The NMR spectrum of the isolated mono hydrochloride PIP–TPE·HCl was consistent with protonation at an exterior tertiary nitrogen of the piperazine moiety. Hence, we assumed that PIP–TPEH^+^ would be the actual predominant photoactive species that is observed in lysosomes. We thus decided to use it in our computational studies, and will henceforth refer to it in short as PIP–TPEH^+^_(soln)_. On the other hand, for the calculations of PIP–TPE in the bulk, we took crystal clusters to consist exclusively of neutral PIP–TPE molecules, and assumed a neutral PIP–TPE molecule imbedded in such a cluster to be the photoactive species. We will thus use PIP–TPE_(bulk)_ to refer to the QM PIP–TPE molecule in such a cluster in all further discussions of our computational results.

**Fig. 7:**
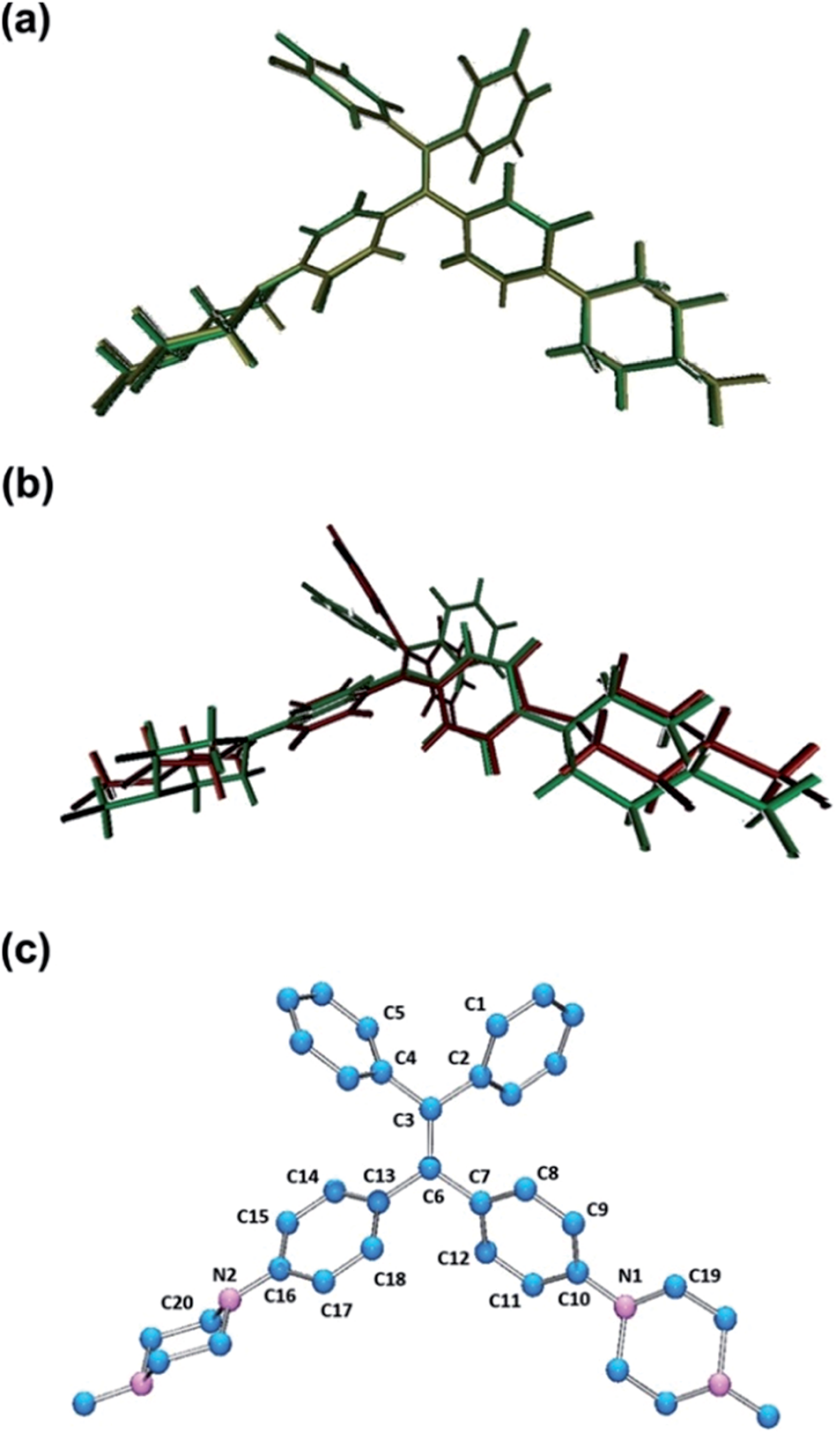
Overlays of (a) ground state (S_0_) optimized structures of PIP–TPE and PIP–TPEH^+^_(soln)_ (yellow = PIP–TPE; green = PIP–TPEH^+^_(soln)_) and (b) ground state (S_0_) and first excited state (S_1_) optimized structures of PIP–TPEH^+^_(soln)_ (green = S_0_; red = S_1_); (c) labeling scheme for PIP–TPE and related species.

To explore if and how protonation and/or subsequent photoexcitation might influence our assumed photoactive species in question, we performed geometry optimizations of PIP–TPE and PIP–TPEH^+^_(soln)_ in the ground state and then geometry optimization of PIP–TPEH^+^_(soln)_ in the first excited state, and studied any major changes in certain relevant metric parameters of these molecules (see Fig. 7 and Table 1).

**Table 1:** Metric parameters of PIP–TPE and PIP–TPEH^+^_(soln)_ in the ground state (S_0_) and first excited state (S_1_). For the labeling scheme see Fig. 7c. Bond lengths (*d*) are given in Å, and angles in °

Metric parameter	PIP–TPE	PIP–TPEH^+^ in S_0_	PIP–TPEH^+^ in S_1_
*d*(C2–C3)	1.492	1.491	1.463
*d*(C3–C4)	1.492	1.492	1.472
*d*(C3–C6)	1.368	1.367	1.485
*d*(C6–C7)	1.487	1.488	1.436
*d*(C7–C8)	1.402	1.407	1.428
*d*(C8–C9)	1.393	1.387	1.378
*d*(C9–C10)	1.410	1.413	1.421
*d*(C10–C11)	1.413	1.409	1.419
*d*(C11–C12)	1.388	1.394	1.383
*d*(C12–C7)	1.407	1.402	1.425
*d*(C6–C13)	1.488	1.490	1.446
*d*(C13–C14)	1.407	1.401	1.466
*d*(C14–C15)	1.387	1.394	1.522
*d*(C15–C16)	1.412	1.405	1.513
*d*(C16–C17)	1.409	1.408	1.512
*d*(C17–C18)	1.394	1.398	1.525
*d*(C18–C13)	1.402	1.407	1.458
*d*(C10–N1)	1.407	1.408	1.389
*d*(C16–N2)	1.409	1.420	1.414
∠(C2–C3–C4)	115.03	115.10	124.04
∠(C7–C6–C13)	115.27	115.08	123.74
∑ of angles around N1	346.94	345.29	351.95
∑ of angles around N2	346.19	344.78	345.86
*τ* (C1–C2–C3–C6)	133.75	–133.35	–156.06
*τ* (C5–C4–C3–C6)	–133.61	–133.56	–151.93
*τ* (C3–C6–C13–C14)	47.04	49.23	23.87
*τ* (C3–C6–C7–C12)	–135.40	–135.81	–157.46
*τ* (C9–C10–N1–C19)	–5.61	51.14	40.36
*τ* (C17–C16–N2–C20)	7.38	–55.40	–55.49
*τ* (C2–C3–C6–C7)	24.32	23.44	82.41

The optimized ground state geometry of PIP–TPE obtained at the DFT level of theory reproduces its single crystal X-ray structure well. Ground state geometry optimization of PIP–TPEH^+^_(soln)_ yields a structure that displays no pronounced changes in the metrics in comparison to the original PIP–TPE (see Fig. 7a) except for a meaningless flip of the protonated piperazine ring (*τ* (C9–C10–N1–C19)). However, expectedly marked changes in bond lengths, angles and dihedral angles of the TPE-fragment and protonated piperazine moiety in PIP–TPEH^+^_(soln)_ upon photoexcitation are predicted (Fig. 7b). Distinct changes in the dihedral angles and in the bond lengths of TPE phenyl rings (see Table 1 and Fig. 7b) as well as a slight planarization of the nitrogen N1 of the protonated piperazine moiety are expected (see Table 1). Also, a remarkable increase of the dihedral angle *τ* (C2–C3–C6–C7) from 23.44° in the ground state to 82.41° in the first excited state is anticipated, indicating that in the excited state the C=C bond of the TPE-fragment is significantly more twisted. Furthermore, in the excited state this C=C double bond is also significantly elongated and loses its “double bond” character upon excitation. Based on these predictions, one might speculate that this twisting around the C3=C6 double bond in the excited state of PIP–TPEH^+^_(soln)_ as well as the rotation of the phenyl groups and piperazine moieties are all possible relaxation channels that are responsible for the radiationless decay in non-viscous media but get blocked in the more viscous lysosomal environment causing the fluorescence to turn on (*i.e.* to be visible to the naked eye).^49^

**Fig. 8:**
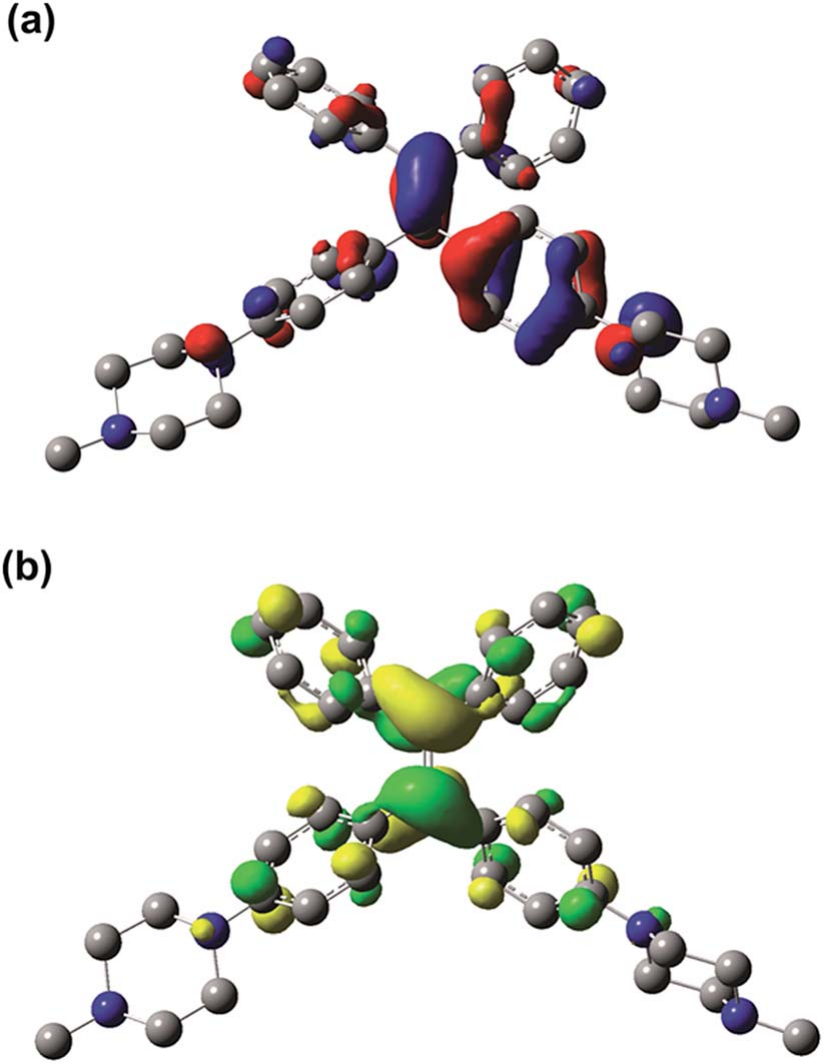
(a) HOMO and (b) LUMO for PIP–TPEH^+^_(soln)_ (*iso* value of 0.04); hydrogen atoms are omitted for clarity.

The results of the TD-DFT calculations for the first excited state (S_1_) of PIP–TPEH^+^_(soln)_ show that it is dominated by a single excitation from the HOMO to the LUMO in this molecule. Both the HOMO and the LUMO in PIP–TPEH^+^_(soln)_ are located mainly on the carbon atoms of the C=C double bond contributing the most with an additional less substantial contribution from the four phenyl rings of the TPE-moiety (see Fig. 8 and S13†). Strikingly, the calculations further indicate that the lone pairs of the aniline nitrogen atoms in PIP–TPEH^+^_(soln)_ are an integral part of the HOMO but not the LUMO. We further note that the same holds true for the HOMO and LUMO distributions of PIP–TPE and PIP–TPE_(bulk)_ (see Fig. S12 and S14 in the ESI†). This means that the molecule has a weak D–A structure, in which the aniline nitrogen atoms act as donors, while the TPE moiety is an acceptor. Thus, we speculate that the observed shift in fluorescence emission (from 404 to 430 nm) for PIP–TPE in solvents of different polarity (*vide supra*) is caused by a twisted intramolecular charge transfer (TICT) process.^35^ However, no twisting is expected for PIP–TPE_(bulk)_, an assumption that is substantiated by the results of the calculations showing that the optimized ground state (S_0_) and first excited state (S_1_) geometries of PIP–TPE_(bulk)_ both have a dihedral angle *τ* (C2–C3–C6–C7) similar to the one calculated for PIP–TPE (Table 1 and Fig. S11b†). We further note that a comparison of the optimized ground state (S_0_) and first excited state (S_1_) geometries of PIP–TPE_(bulk)_ reveals only negligible differences in the metrics of these structures (see Fig. S11b†). Thus, the deep blue emission (λ_PL,max_ = 410–419 nm) observed for PIP–TPEH^+^_(soln)_ in polar milieu can be attributed to the higher twisting degree of the molecule in the excited state that would automatically result in less efficient conjugation, while the red-shifted yellowish-green emission (λ_PL,max_ = 493 nm) of PIP–TPE_(bulk)_ could be attributed to the higher degree of conjugation preserved in the excited state of the molecule upon excitation.^50^,^51^ Furthermore, TD-DFT (B3LYP:UFF) calculations for PIP–TPE_(bulk)_ result in a λ_PL,max_ = 471 nm that is in good agreement with experiment (λ_PL,max_ = 493 nm) and appears to confirm the proposed model.

## Experimental

### Materials

All chemicals and reagents were used as received from Sigma-Aldrich without further purification unless otherwise specified. Minimum essential medium (MEM), Dulbecco’s modified Eagle’s medium (DMEM), phosphate buffered saline (PBS), fetal bovine serum (FBS), penicillin and streptomycin, LysoTracker® Red DND-99, and (3-(4,5-dimethylthiazol-2-yl)-2,5-diphenyltetrazolium bromide) were purchased from Invitrogen. Buffer solutions (pH = 1–13) were purchased from Fisher Scientific. Chloroquine was purchased from Bide Pharmatech Ltd. Milli-Q water was supplied by Milli-Q Plus System (Millipore Corporation, United States).

### Characterization, measurement and instruments

The ^1^H and ^13^C NMR spectra were measured on a Bruker ARX 400 NMR spectrometer using CDCl_3_ as the solvent and tetramethylsilane (TMS: *δ* = 0 ppm) as an internal standard. High-resolution mass spectra (HRMS) were recorded on a Finnigan MAT TSQ 7000 Mass Spectrometer System operating in MALDI-TOF mode. Suitable single crystals of PIP–TPE were selected under oil under ambient conditions. Single crystal X-ray diffraction intensity data were collected in a stream of cold nitrogen at 100 K on a SuperNova, Dual, Mo at zero, Atlas diffractometer. Using Olex2,^52^ the structure was solved with the ShelXS^53^ structure solution program using Direct Methods and refined with the ShelXL^54^ refinement package using Least Squares minimisation. The details concerning X-ray crystallographic structure solutions and refinement for PIP–TPE are tabulated in Table S1.† The UV-vis absorption spectra were obtained using a UV-vis spectrometer (Shimadzu, UV-2600, Japan). The PL measurements were carried out on a Horiba Fluoromax-4 spectrofluorometer. Particle sizes were measured on a Zeta potential analyzer (Brookhaven, ZETAPLUS). The absolute fluorescence quantum yields were measured using a Hamamatsu quantum yield spectrometer C11347 Quantaurus_QY. The viscosities of the methanol–glycerol mixtures were measured using a TA ARES-G2 (serial: 4010-0538) rotational rheometer. Fixed Cell imaging of PIP–TPE in HeLa cells was collected on a BX41 Fluorescence Microscope. Confocal images were collected on a Zeiss laser scanning confocal microscope (LSM7 DUO) and analyzed using ZEN 2009 software (Carl Zeiss). All calculations reported herein were performed using the Gaussian 09 package.^41^ Density functional theory (DFT) was employed with a 6-31G (d) basis set of B3LYP for full geometry optimization.

### Synthesis of the compound PIP–TPE

The starting material 1,1′-(2,2-diphenylethenylidene)bis(4-bromo)-benzene was synthesized according to the procedure published by Li *et al.*^55^ In a 100 mL round-bottom 2-neck flask, 0.449 g (1.55 mmol) tri-*tert*-butylphosphonium tetrafluoroborate (P(*t*Bu)_3_HBF_4_), 3.600 g (11.00 mmol) cesium carbonate (Cs_2_CO_3_), and 20 mL dry and degassed toluene were added and stirred for 1 h at r.t. under nitrogen. Then 0.097 g (0.43 mmol) Pd(OAc)_2_ was added. The reaction mixture was stirred for another 0.5 h under N_2_. Upon the formation of an orange suspension, 1.000 g (2.04 mmol) 1,1′-(2,2-diphenylethenylidene)bis(4-bromo)-benzene and 0.5 mL (4.51 mmol) of 1-methylpiperazine were added, and a yellowish green suspension formed immediately. The reaction mixture was allowed to reflux under N_2_ for 2 days. After the reaction was cooled down to r.t., the toluene was removed; 50 mL EtOH and 10 mL acetone were added to dissolve the crude product. The suspension was filtered, concentrated under reduced pressure and purified *via* silica gel flash chromatography (chloroform/MeOH = 40 : 1). 0.162 g of yellow powder of PIP–TPE was obtained. Yield: 15%. Pale yellow block crystals were grown from acetone/hexane *via* solvent diffusion. ^1^H NMR (400.132 MHz, CDCl_3_), *δ*_H_ (TMS, ppm): 7.12–6.99 (m, 10H, Ar–*H*), 6.94–6.87 (m, 4H, Ar–*H*), 6.67–6.59 (m, 4H, Ar–*H*), 3.25–3.04 (m, 8H, N–C*H*_*2*_), 2.59–2.42 (m, 8H, N–C*H*_*2*_), 2.32 (s, 6H, Me–*H*). ^13^C NMR (100.632 MHz, CDCl_3_), *δ*_C_ (TMS, ppm): 149.27, 144.72, 140.51, 138.28, 135.08, 132.40, 131.46, 127.63, 125.83, 114.58, 55.15, 48.60, 46.22, 11.20. MALDI-TOF-MS: *m*/*z* calcd for [C_36_H_40_N_4_]: 528.33; found 528.3274.

### The protonation of the compound PIP–TPE

In a 4 mL screw top vial, PIP–TPE (11.2 mg, 0.02 mmol) was dissolved in dichloromethane (2 mL) to form a yellow solution. Hydrochloric acid (36.5–38.0 wt%, 0.1 mL, 1.21 mmol) was added and a white suspension formed immediately. After stirring for a few seconds, the suspension dissolved and the color of the solution changed to pale yellow. The vial was capped and the reaction mixture was stirred overnight. The next day, the layer of dichloromethane solution was pipetted out and the remaining aqueous layer was extracted with dichloromethane (10 times with 2 mL). Then, all dichloromethane layers were combined and after rotoevaporation, a pale yellow powder of the protonated PIP–TPE was obtained (10.0 mg). Yield: 89%. ^1^H NMR (400.132 MHz, CDCl_3_), *δ*_H_ (TMS, ppm): 12.99 (broad, N*H*^+^), 7.15–7.05 (m, 6H, Ar–*H*), 7.04–6.96 (m, 4H, Ar–*H*), 6.96–6.82 (m, 4H, Ar–*H*), 6.81–6.53 (m, 4H, Ar–*H*), 3.91–3.28 (m, 12H, N–C*H*_*2*_), 3.26–2.97 (m, 4H, N–C*H*_*2*_), 2.86 (s, 6H, Me–*H*). MALDI-TOF-MS: *m*/*z* calcd for [C_36_H_40_N_4_H^+^]: 529.33; found 529.3319.

### Viscosity measurements

The viscosities of the aqueous buffer–glycerol mixtures were measured using a TA ARES-G2 (serial: 4010-0538) rotational rheometer at a temperature of 25 ± 0.5 °C. Each measurement used *ca.* 1 mL of each sample. The viscosity was measured as a function of shear rate in the range from 100.0 to 0.01 s^–1^. In Fig. 4C and S7,† we adjust the ratio of aqueous buffer and glycerol solution and achieve different viscosity mixtures (2.15 cP–438.40 cP at 25 °C, 1 cP = 1 mPa s). The ratios are as follows: 2.15 cP at 25 °C (3120 μL aqueous buffer : 800 μL glycerol), 7.94 cP at 25 °C (1920 μL aqueous buffer : 2000 μL glycerol), 32.46 cP at 25 °C (1120 μL aqueous buffer : 2800 μL glycerol), 78.90 cP at 25 °C (740 μL aqueous buffer : 3200 μL glycerol), 140.60 cP at 25 °C (520 μL aqueous buffer : 3400 μL glycerol), 246.70 cP at 25 °C (320 μL aqueous buffer : 3600 μL glycerol), and 438.40 cP at 25 °C (120 μL aqueous buffer : 3800 μL glycerol). The viscosity results are shown in Fig. S7.†

### Cell culture and cell imaging

HeLa cells were cultured in MEM and DMEM, respectively. All the cells were cultured in media supplemented with 10% heat-inactivated FBS, 100 units per mL penicillin and 100 μg mL^–1^ streptomycin, in a humidity incubator with 5% CO_2_ at 37 °C. Before experiment, the cells were pre-cultured until confluence was reached.

All the cells were grown overnight on a 35 mm Petri dish with a cover slip or a plasma-treated 25 mm round cover slip mounted to the bottom of a 35 mm Petri dish with an observation window. The live cells were incubated with 1 μM PIP–TPE (2 μL of a 1 mM stock solution of PIP–TPE in DMSO was diluted to 2 mL culture medium) for 15 min, and then were imaged under fluorescence microscopy. For co-staining experiments, the cells were co-stained with PIP–TPE (1 μM) and a commercial biomarker (LysoTracker Red DND-99) for 15 min. The cells were then imaged using a laser-scanning confocal microscope (LSM7 DUO) using a 405 nm and 561 nm laser as the excitation light. The spectral collection region was 425–545 nm and 575–625 nm, respectively.

### Cell viability evaluated by MTT assay

The HeLa cells were seeded in 96-well plates at a density of 5000 cells per well. After overnight culture, the medium in each well was replaced by fresh medium containing different concentrations (0.1/0.2/0.5/1/2/5 μM) of PIP–TPE. After 24 h of treatment, 10 μL MTT solution (5 mg mL^–1^ in phosphate buffer solution) was added into each well. After incubation for 4 h at 37 °C, the remaining medium in each well was totally removed and then replaced by 50 μM DMSO. With mixing for 15 min, the absorbance of each well at 595 nm was recorded by the plate reader (Thermo Scientific™ Varioskan™ LUX multimode microplate reader). Each experiment was performed at least 6 times as parallel tests.

### Lysosome trace tracing

For the lysosome trace tracking experiment, the cells were first incubated with 1 μM PIP–TPE for 15 min and then treated with 3 μM chloroquine. The images at different points in time were recorded using a confocal microscope with an excitation wavelength of 405 nm. The merged images were analysed using the software ZEN 2009.

## Conclusions

In conclusion, we have developed an acidic pH independent lysosome targetable AIEgen, PIP–TPE, which displays fluorescence emission of different colors depending on its aggregation state: deep-blue for non-aggregates and yellowish-green for the bulk solid. Experiments suggest that protonation of PIP–TPE has no significant influence on its photophysical properties but does affect its scale of aggregation. PIP–TPE has proved to have high specificity to lysosomes with a good signal-to-noise ratio and negligible cytotoxicity. PIP–TPE’s good selectivity towards lysosomes can be attributed to the piperazine functional groups and higher viscosity of the intralysosomal milieu and renders it a good lysosomal tracing agent with better photostability than the commercial alternatives such as LysoTracker Red. PIP–TPE’s turn-on fluorescence in lysosomes can be attributed to the viscosity restricting intramolecular motion (RIM). PIP–TPE’s blue emission in lysosomes can be attributed to the high degree of C=C twisting of its protonated species in the excited state upon excitation. Based on the aforementioned facts, this molecular design can be used as a guide to develop new promising lysosomal pH stable fluorescent AIE probes, which would allow monitoring of some important fundamental microenvironmental parameters with no or little influence from pH.

## Conflicts of interest

There are no conflicts to declare.

## Supplementary Material

Supplementary information

Crystal structure data
