# Supplementary material for: An acidic pH independent piperazine–TPE AIEgen as a unique bioprobe for lysosome tracing
Source: Chem Sci. 2017 Sep 18;8(11):7593–603. doi: 10.1039/c7sc03515b (PMC5848814; doi:10.1039/c7sc03515b)
Supplement: Supplementary file 1 [file SC-008-C7SC03515B-s001.pdf]

Electronic Supplementary Material (ESI) for Chemical Science.
This journal is © The Royal Society of Chemistry 2017

Electronic Supplementary Information (ESI)

1. NMR spectra of **PIP-TPE** and VT-NMR spectra of **protonated PIP-TPE**

2. Single Crystal X-ray Crystallography of **PIP-TPE**

3. Confocal images of **PIP-TPE** in H_2O

4. Fluorescence quantum yields of **PIP-TPE** in MeOH with different fractions of glycerol

5. Particle sizes of **PIP-TPE** in aqueous buffer solutions

6. Viscosity measurement

7. Live cell and Fixed cell fluorescent imaging of **PIP-TPE** in Hela cells

8. MTT of **PIP-TPE**

9. Photostability of **PIP-TPE**

10. Theoretical calculations of **PIP-TPE**, protonated **PIP-TPE** and **PIP-TPE** in bulk

1. NMR spectra of PIP-TPE and VT-NMR spectra of protonated PIP-TPE

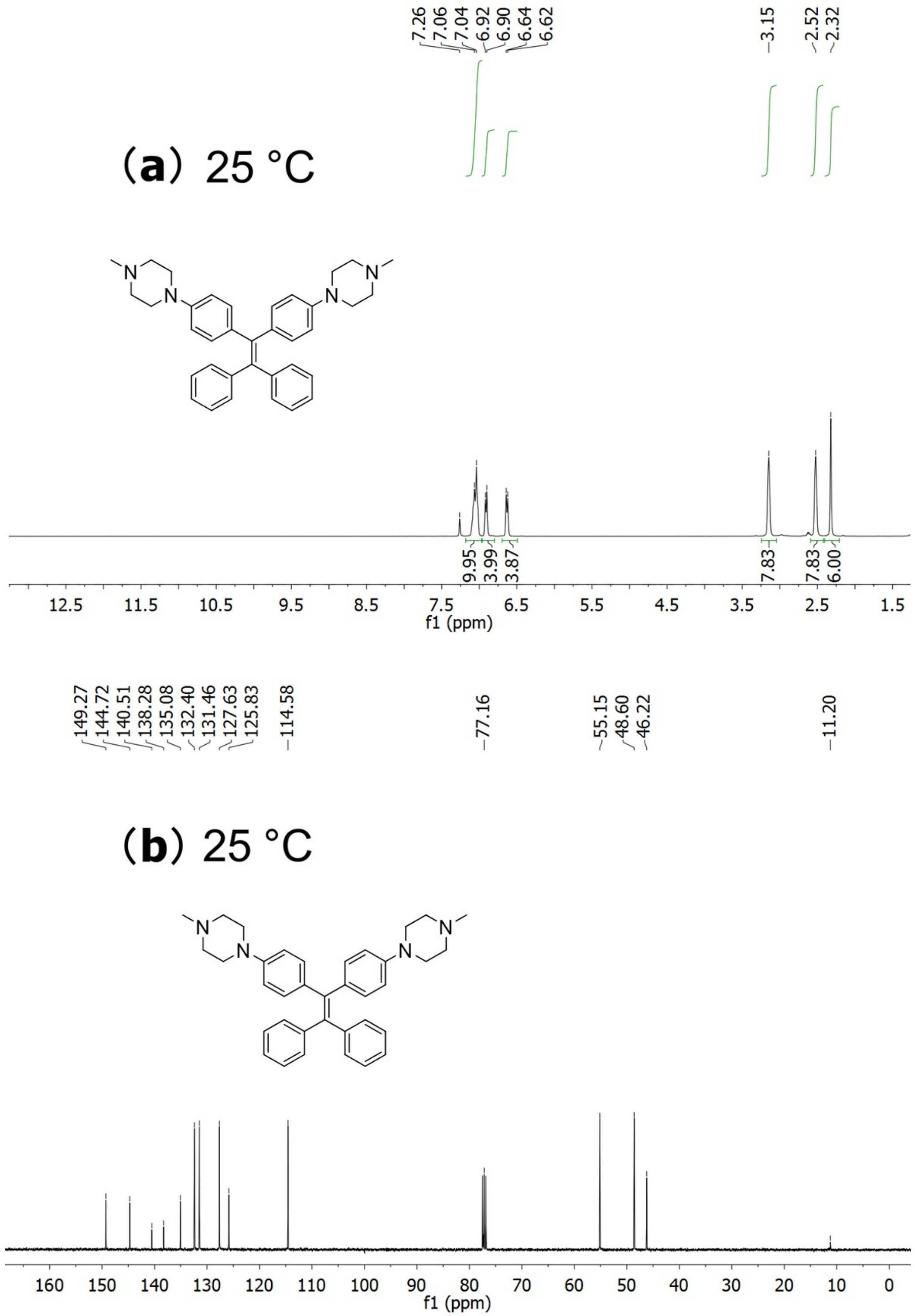

Figure S1 (a) ^{1}H-NMR (400.132 MHz, $CDCl_3$/TMS) and (b) ^{13}C-NMR (100.632 MHz, $CDCl_3$/TMS) of **PIP-TPE**

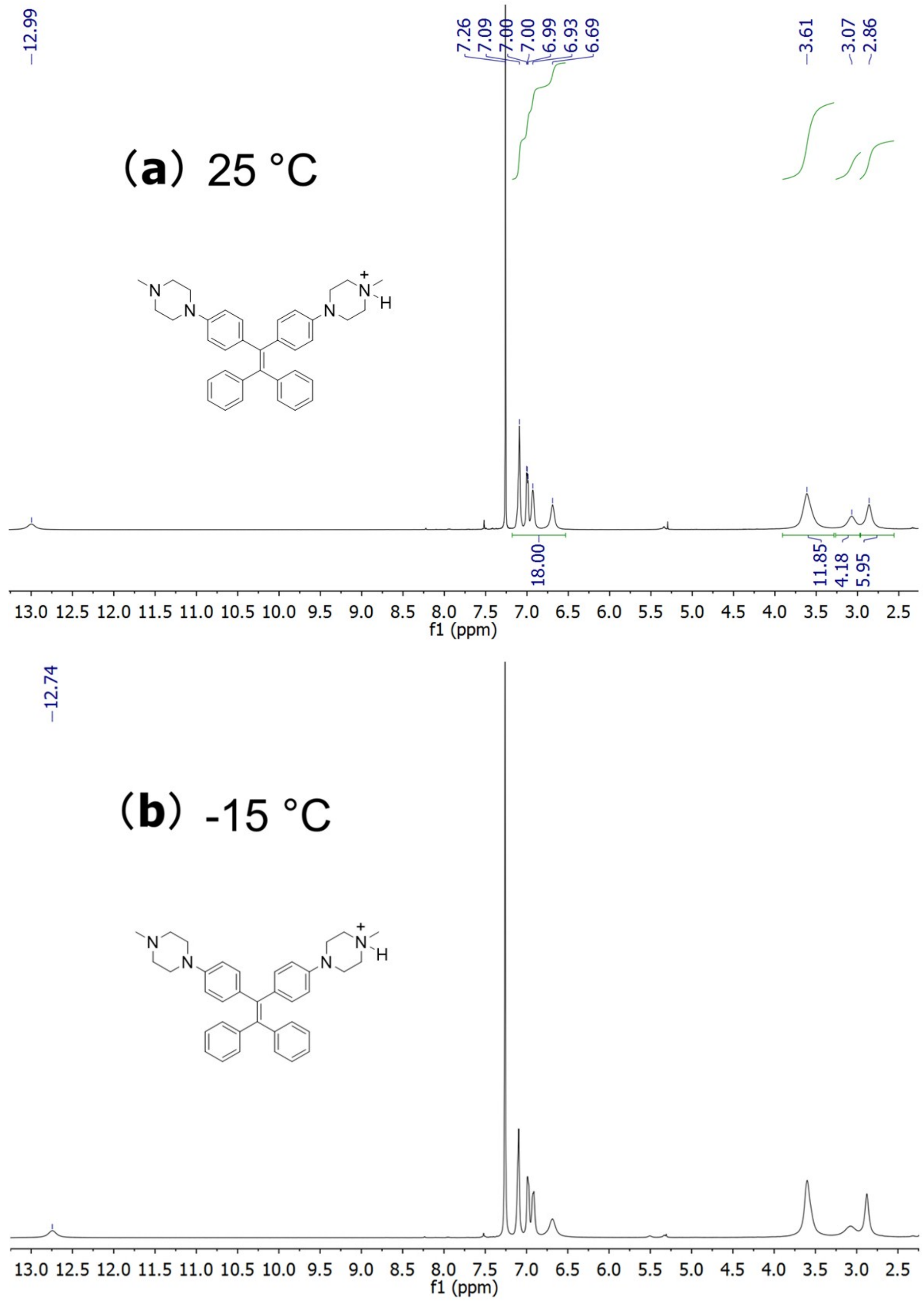
(a) 25 °C
12.99
7.26
7.09
7.00
7.00
6.99
6.93
6.69
3.61
3.07
2.86
18.00
11.85
4.18
5.95
13.0 12.5 12.0 11.5 11.0 10.5 10.0 9.5 9.0 8.5 8.0 7.5 7.0 6.5 6.0 5.5 5.0 4.5 4.0 3.5 3.0 2.5
f1 (ppm)
(b) -15 °C
12.74
13.0 12.5 12.0 11.5 11.0 10.5 10.0 9.5 9.0 8.5 8.0 7.5 7.0 6.5 6.0 5.5 5.0 4.5 4.0 3.5 3.0 2.5
f1 (ppm)

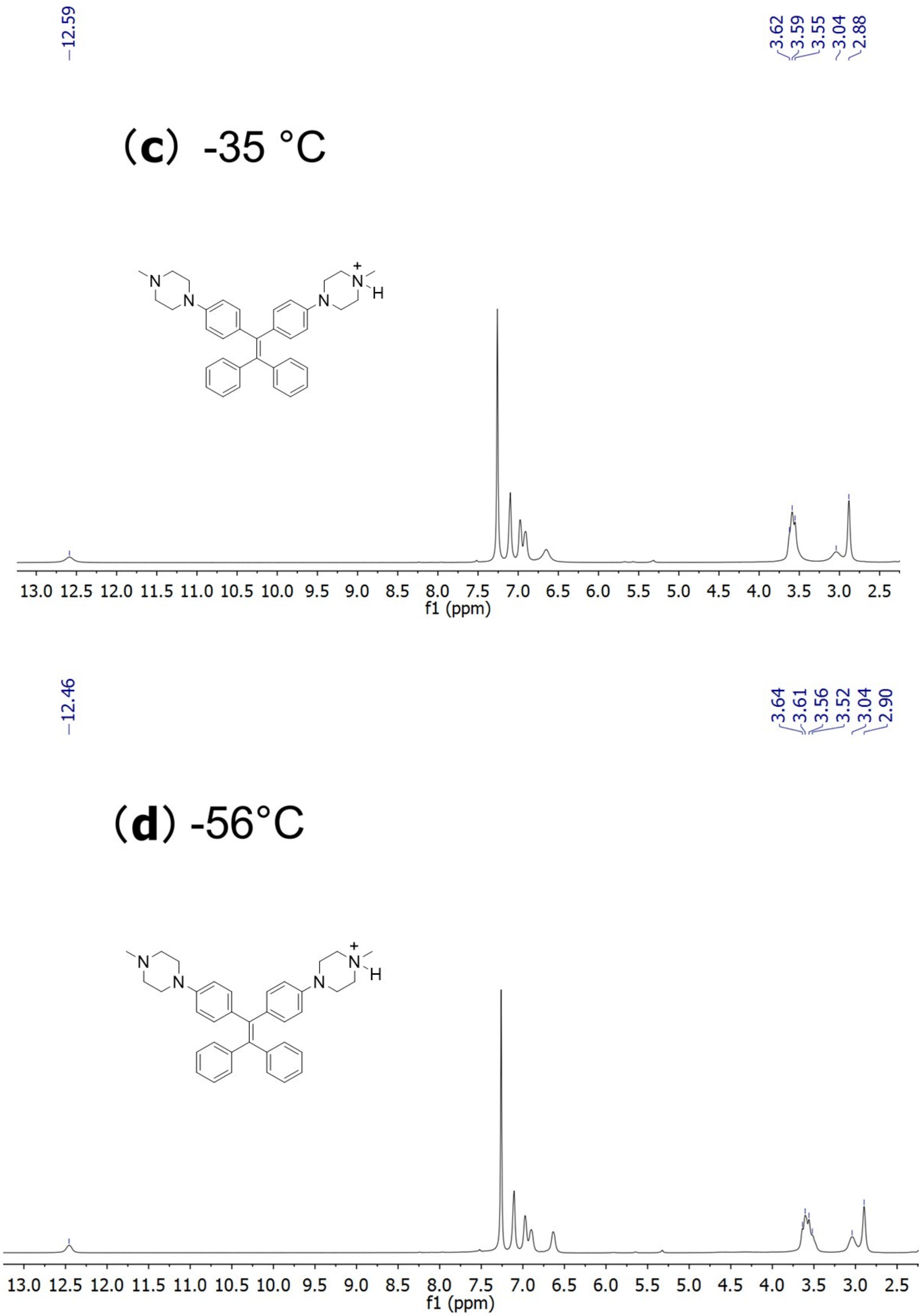

Figure S2 VT-^{1}H NMR of protonated **PIP-TPE** (400.132 MHz, $CDCl_3$)

2. Single Crystal X-ray Crystallography of PIP-TPE

Table S1 The crystallographic details of the compound **PIP-TPE**

Compound	**PIP-TPE**
CCDC No.	1555412
empirical formula	$C_{36}H_{40}N_4$
formula weight	528.72
crystal dimensions, mm	0.25 × 0.1 × 0.08
crystal system	monoclinic
space group	$P2_1/c$
a/Å	13.1123(13)
b/Å	20.5840(16)
c/Å	11.2600(9)
α/°	90
β/°	99.391(9)
γ/°	90
Volume/Å^3	2998.4(5)
Z	4
ρ_{calc}, mg/mm^3	1.171
F(000)	1136.0
λ/ Å	MoKα (λ = 0.71073)
μ/mm $^{-1}$	0.069
temperature/K	100.00(10)
2θ range for data collection	6.544 to 51.992
reflections collected	17830
independent reflections. (R_{int})	5841 (0.1229)
data/restraints/parameters	5841/0/363
goodness-of-fit on F^2	1.000
R_1,wR_2 [I>=2σ (I)]	0.0679, 0.0990
R_1,wR_2 [all data]	0.1569, 0.1222
largest diff. peak/hole / e Å^{-3}	0.20/-0.23

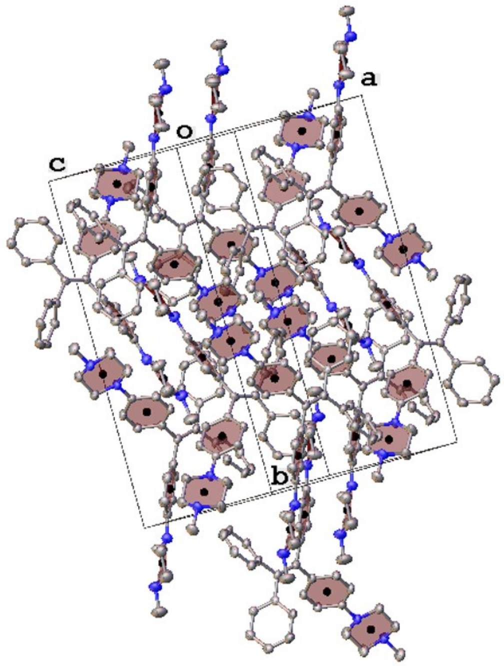

Figure S3 Crystal packing of **PIP-TPE** (piperazines and their neighboring phenyls are highlighted in red)

3. Confocal images of PIP-TPE in H_2O

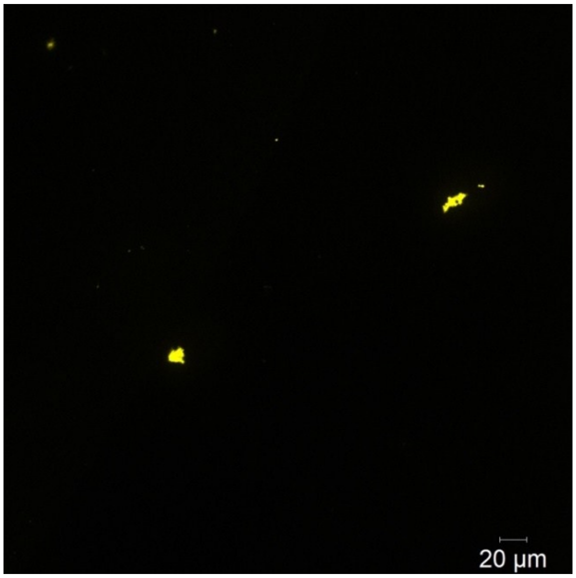

Figure S4 Confocal image of **PIP-TPE** as amorphous particles in H_2O (50 μM), Scale bar = 20 μm.

4. Fluorescence quantum yields of PIP-TPE in MeOH with different fractions of glycerol

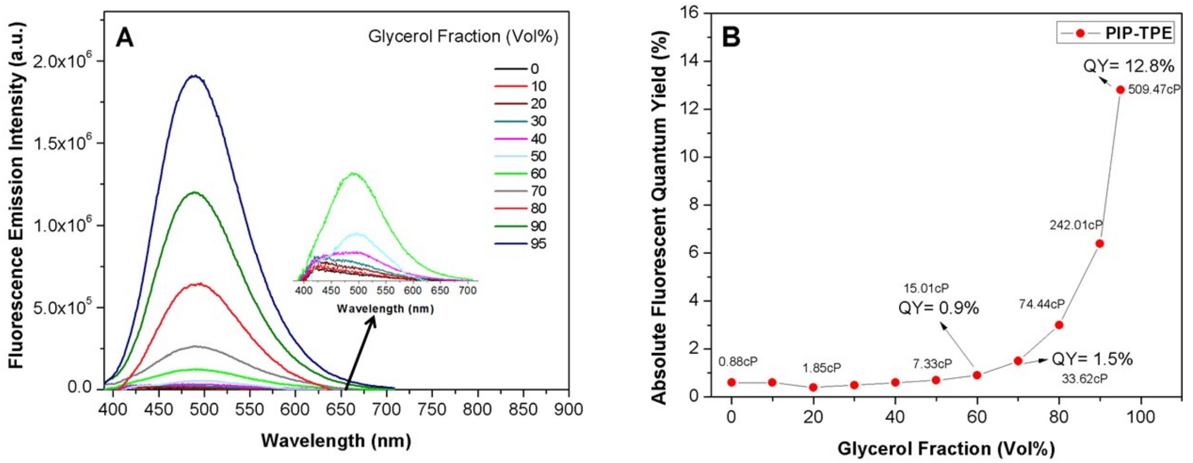

Figure S5 Photoluminescent intensity PL (A) and absolute fluorescence quantum yields $Ø_{PL}$ (B) of **PIP-TPE** (10^{-5}M) in MeOH with different fractions of glycerol; all data were collected under λ_{ex} = 360nm at 25 °C.

5. Particle sizes of PIP-TPE in aqueous buffer solutions

The Particle sizes of **PIP-TPE** in aqueous buffer solutions were measured on a Zeta potential analyzer (Brookhaven, ZETAPLUS).

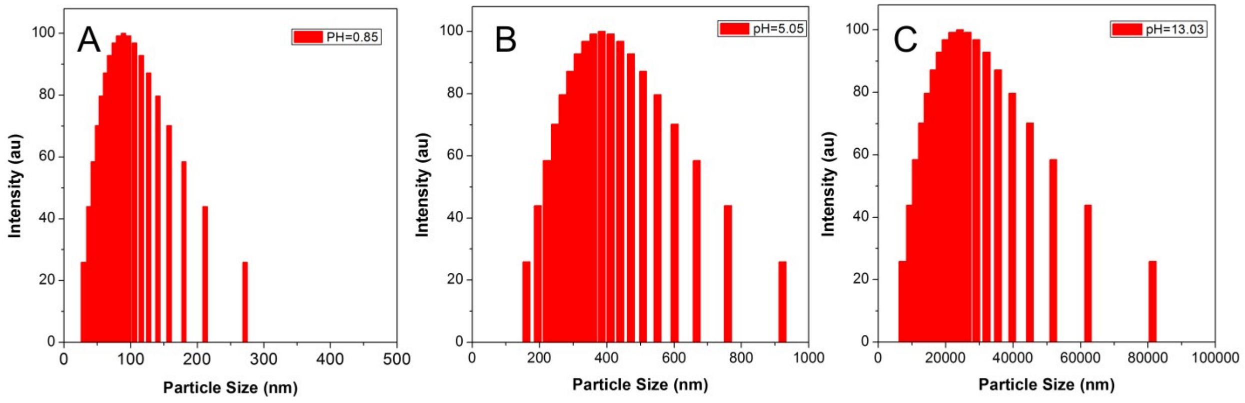

Figure S6 The particle sizes of **PIP-TPE** in aqueous buffer solutions of pH 0.85, 5.05 and 13.03

(0.8×10^{-5}M) are 89 nm, 385 nm and 24.2μm, respectively.

6. Viscosity measurement

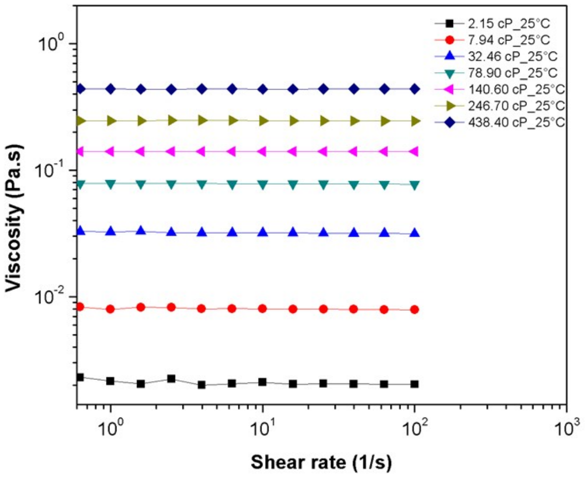

Figure S7 The viscosity of aqueous buffer-glycerol mixtures at 25±0.5°C.

7. Live cell and Fixed cell fluorescent imaging of PIP-TPE in Hela cells

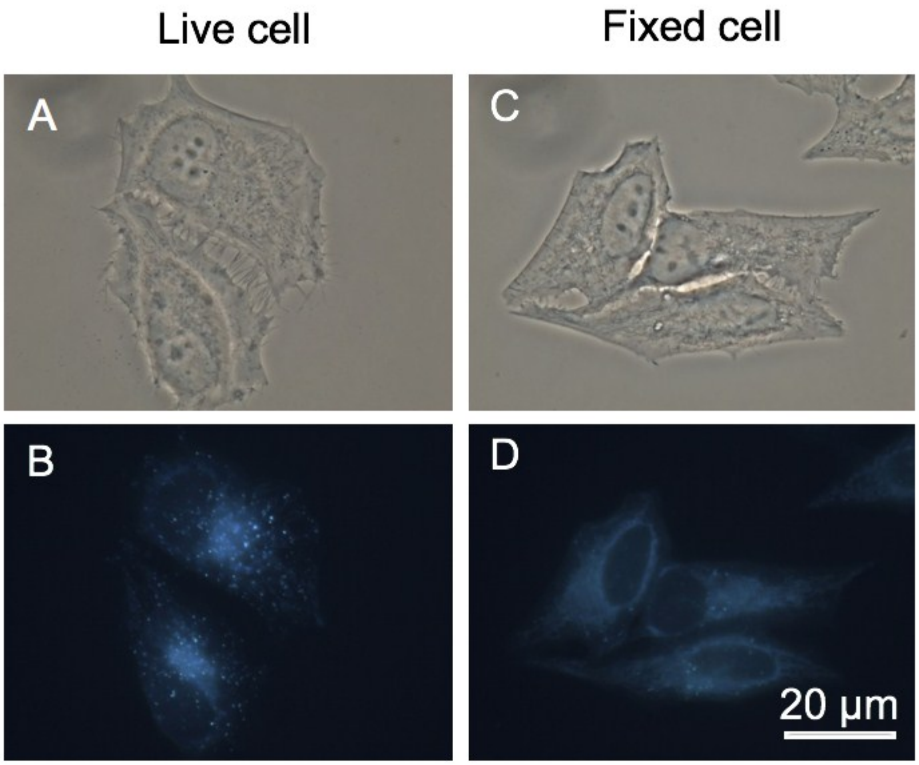

Figure S8 (A and C) Bright field and (B and D) fluorescent images of HeLa cells incubated with 1 μM **PIP-TPE** for 15min then (A-B) without treatment or (C-D) with 2mL 4% PFA (paraformaldehyde) treatment for 15 min. Excitation wavelength: 330-385 nm; Scale bar = 20 μm.

8. MTT of PIP-TPE

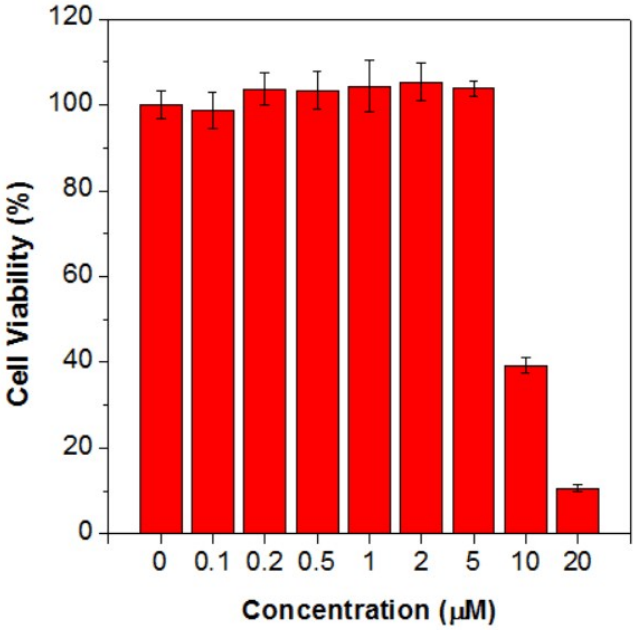

Figure S9 Cell viability of HeLa cells after incubation with different concentrations of **PIP-TPE**.

9. Photostability of PIP-TPE

For the photostability test, the cells were imaged by a confocal microscope (Zeiss Laser Scanning Confocal Microscope; LSM7 DUO) using ZEN 2009 software (Carl Zeiss). **PIP-TPE** was excited at 405 nm (2% laser power) and LysoTracker Red was excited at 560 nm (2% laser power).

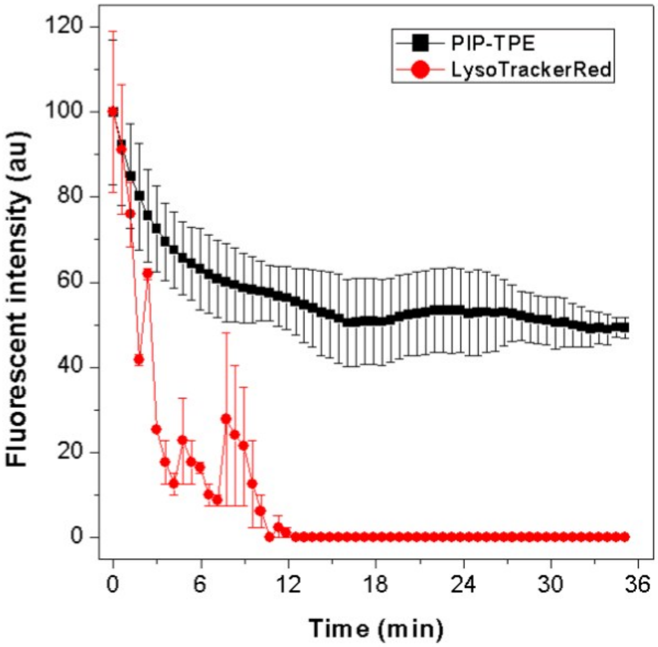

Figure S10 The photostability of **PIP-TPE** and LysoTracker Red

10. Theoretical calculations of PIP-TPE, protonated PIP-TPE and PIP-TPE in bulk

Quantum mechanical calculations for the **PIP-TPE** single molecule in solution were performed at the DFT level of theory using B3LYP functional [1-4] and 6-31G(d) basis set as implemented in the D0.1 version of the Gaussian 09 software package. [5] The bulk solvent (water) effect has been modeled using the polarizable continuum model (PCM). [6] Possible static correlations in the system were modeled using Grimme's density functional empirical dispersion corrections as implemented in the D0.1 version of the Gaussian 09. [7] The equilibrium solvation method was employed for geometry optimization and vibrational frequency calculations. The solution-phase Hessian of S_0 was evaluated analytically at the DFT level indicated above and had no negative eigenvalues.

The photophysical properties of **PIP-TPE** in the aggregated state were analyzed using ONIOM(QM:MM) approach, an integrated quantum mechanics: molecular mechanics (QM:MM) method.[8-11] In the two-layer ONIOM calculation the system was divided into two parts, a selected centrally located **PIP-TPE** molecule was treated by a DFT/TD-DFT (QM) method using B3LYP functional[2-4, 12] while the packing surroundings in the crystal (40 **PIP-TPE** molecules) were treated by a low-level method, molecular mechanics (MM) method, the

Universal Force Feld (UFF).[13] Geometry optimization, vibrational frequency and excited state calculations were performed using the 6-31G(d) basis set as implemented in the D0.1 version of Gaussian 09 software package. The solid-phase Hessian of S_0 was evaluated analytically at the DFT level, while that of S_1 was calculated numerically at the TD-DFT level. The electrostatic interactions in the model were calculated semiclassically incorporating the MM charges into the QM Hamiltonian (electronic embedding). [11,14] In our QM/MM model we neglect possible intermolecular excitonic couplings and assume that the intramolecular motions dominate the photophysical process.

During the QM/MM simulations, only the central **PIP-TPE** (QM) molecule was active for optimization, while all the surrounding **PIP-TPE** (MM) molecules remained rigid. No symmetry constraints were imposed during the optimizations in both solution and solid phases. The above PCM and QM/MM methods have been shown to be successful in dealing with varieties of AIEgens with both structure and excited-state decay rates. [15-16]

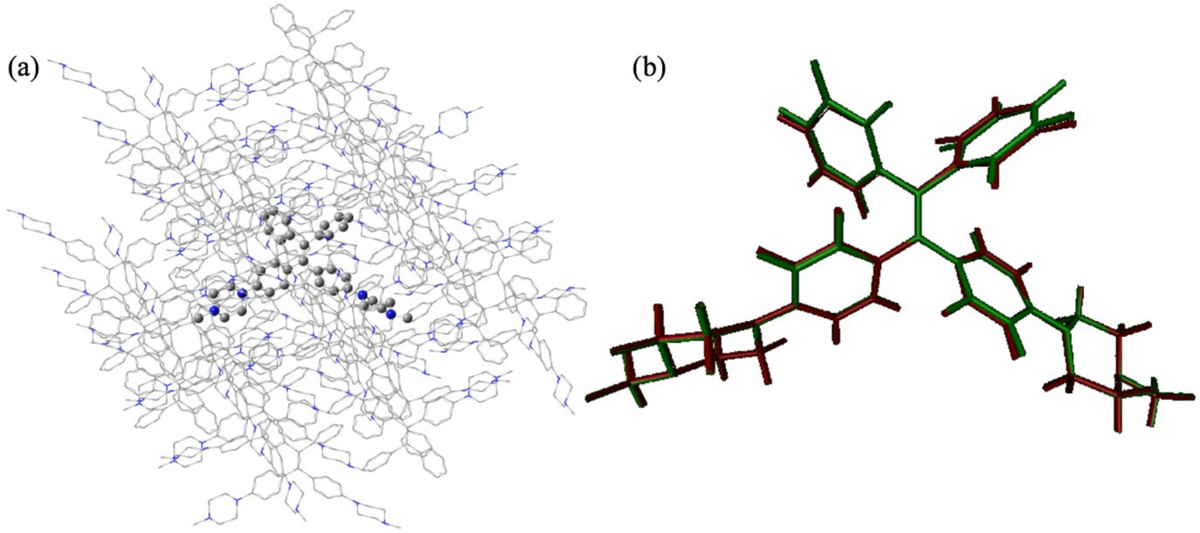

Figure S11 (a) Optimized 40-molecule **PIP-TPE** cluster with **PIP-TPE$_{(bulk)}$** molecule in the ground state calculated using ONIOM QM:MM model of with the high-level part of model shown using ball-and-stick representation, and the low-level part of the model using wire representation; (b) overlay of the optimized ground state (S_0) and first excited state (S_1) structures of **PIP-TPE$_{(bulk)}$** (green = S_0; red = S_1).

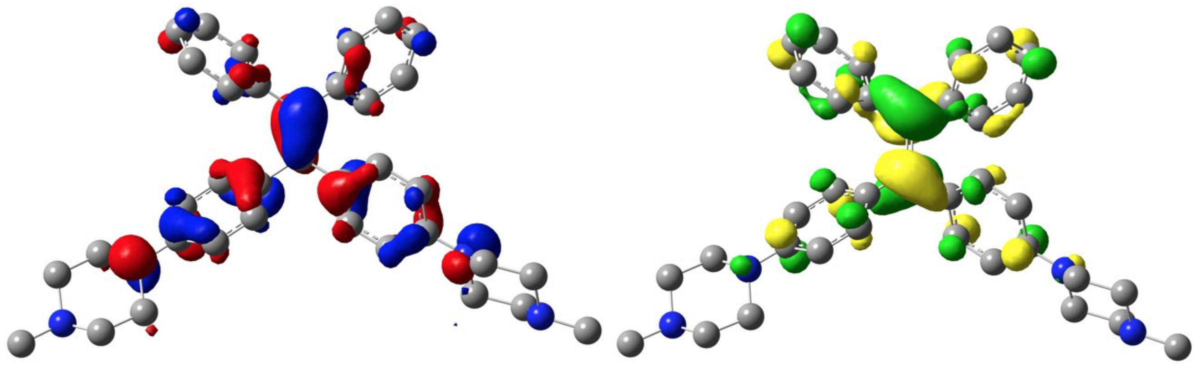

Figure S12 The HOMO (left) and LUMO (right) in **PIP-TPE**. Hydrogen atoms are omitted for clarity. HOMO and LUMO are depicted at *iso* value of 0.04.

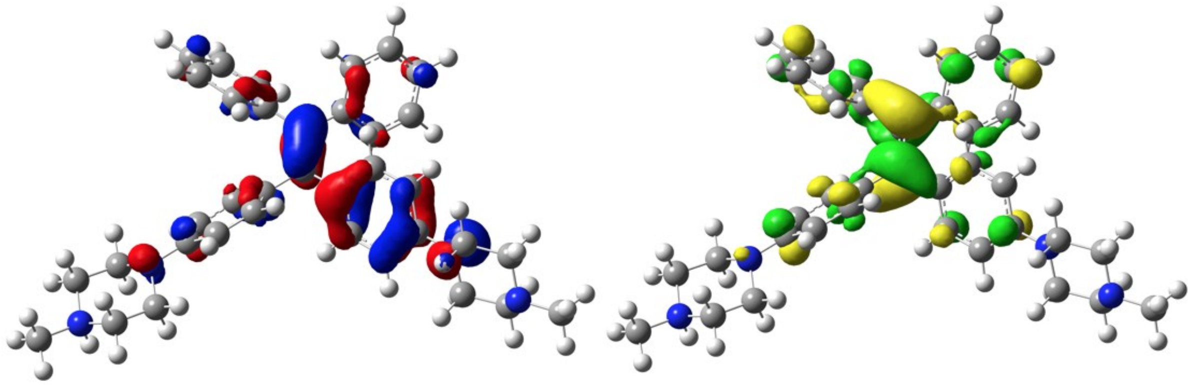

Figure S13 The HOMO (left) and LUMO (right) of **PIP-TPEH**$^{+}_{\mathbf{(soln)}}$; HOMO and LUMO are depicted at *iso* value of 0.04.

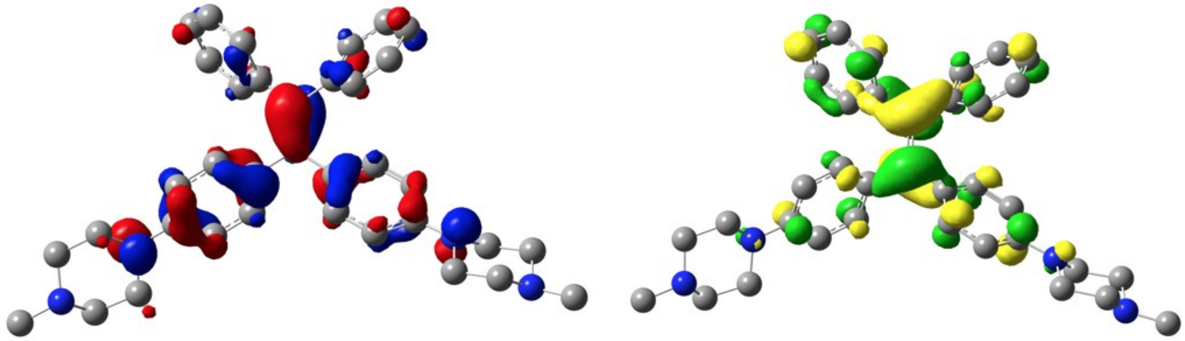

Figure S14 The HOMO (left) and LUMO (right) of **PIP-TPE**$_{\mathbf{(bulk)}}$; Hydrogen atoms are omitted for clarity; HOMO and LUMO are depicted at *iso* value of 0.04.

Optimized Geometries

(1) S_0 geometry of PIP-TPE:

H	8.553326	-4.939947	-0.583412
C	8.441546	-4.156319	0.174012
H	8.553326	-4.939947	-0.583412
H	8.738111	-4.570623	1.143887
H	9.140668	-3.334923	-0.076379
N	7.051193	-3.721223	0.230072
C	6.846716	-2.707599	1.264005
C	5.374553	-2.316616	1.346374
N	4.892658	-1.828556	0.044673
C	3.672087	-1.125318	0.036358
C	3.483932	-0.031768	0.910047
C	2.309771	0.706892	0.894377
C	1.261041	0.406145	0.005610
C	-0.004760	1.189077	-0.003464
C	-0.018872	2.556911	0.003193
C	1.199243	3.370309	-0.278158
C	2.008899	3.100925	-1.395756
H	1.748290	2.272735	-2.047618
C	3.135150	3.876037	-1.668507
H	3.744634	3.651835	-2.540114
C	3.476018	4.941604	-0.830027
H	4.354470	5.545305	-1.041287
C	2.673046	5.230187	0.276998
H	2.926803	6.058516	0.933384
C	1.541667	4.458587	0.543421
H	0.916399	4.692071	1.400792
C	-1.250934	3.345466	0.294852
C	-2.052951	3.051673	1.411873
H	-1.777235	2.221207	2.054668
C	-3.190711	3.805684	1.695555
H	-3.794027	3.562666	2.566421
C	-3.551117	4.874334	0.869098
H	-4.438546	5.461588	1.089019
C	-2.755955	5.187175	-0.236873
H	-3.024681	6.018110	-0.883952
C	-1.613107	4.436525	-0.514296
H	-0.994251	4.689026	-1.370958
C	-1.252163	0.379281	-0.018057

C	-1.401656	-0.740425	0.821281
H	-0.597579	-1.002971	1.503254
C	-2.563251	-1.499963	0.822691
H	-2.651614	-2.322771	1.524927
C	-3.635588	-1.198418	-0.046075
C	-3.474027	-0.098488	-0.913006
H	-4.245300	0.154413	-1.631064
C	-2.312813	0.670171	-0.887857
H	-2.221972	1.511051	-1.568871
N	-4.818729	-1.958166	0.002377
C	-5.960484	-1.509545	-0.792985
H	-5.805736	-1.707138	-1.868555
H	-6.087272	-0.431432	-0.661513
C	-7.238633	-2.206476	-0.328006
H	-7.472130	-1.874804	0.703640
H	-8.068116	-1.892289	-0.971906
N	-7.103251	-3.659892	-0.403443
C	-8.339867	-4.332307	-0.023747
H	-8.216161	-5.416319	-0.123113
H	-9.151268	-4.016327	-0.688596
H	-8.646079	-4.115830	1.018108
C	-5.979969	-4.089850	0.428038
H	-5.870370	-5.177273	0.343959
H	-6.150549	-3.850597	1.496917
C	-4.687603	-3.422388	-0.031993
H	-3.872269	-3.740094	0.618682
H	-4.450487	-3.757665	-1.056803
C	1.441538	-0.691755	-0.847609
C	2.617120	-1.440787	-0.842246
H	2.696321	-2.285604	-1.516523
H	0.643174	-0.969524	-1.530537
H	2.201977	1.546788	1.573930
H	4.277232	0.253970	1.593734
C	5.121309	-2.817040	-1.007973
C	6.598984	-3.202592	-1.059808
H	6.737225	-3.977225	-1.822831
H	7.190359	-2.317834	-1.369918
H	4.840260	-2.383915	-1.972172
H	4.512722	-3.725379	-0.850842
H	4.783092	-3.192818	1.664914
H	5.253407	-1.540458	2.103174
H	7.452675	-1.800133	1.068933
H	7.162448	-3.116505	2.230820

(2) S_0 geometry of PIP-TPEH$^{+}_{(soln)}$:

a. UV-vis calculation:

Excited State 1: Singlet-A 3.2557 eV 380.82 nm f=0.4960 <S**2>=0.000

142 ->143 0.70477

b. Optimized Geometry

H	8.430775	-5.100743	-0.582951
C	8.366107	-4.284555	0.144997
H	8.430775	-5.100743	-0.582951
H	8.684288	-4.667993	1.120683
H	9.078285	-3.494181	-0.161893
N	6.990678	-3.808554	0.229075
C	6.847952	-2.748994	1.226438
C	5.389325	-2.318070	1.344303
N	4.870335	-1.872133	0.041598
C	3.664477	-1.145249	0.045821
C	3.516133	-0.026188	0.895255
C	2.356132	0.734601	0.887010
C	1.281463	0.431941	0.030190
C	0.029691	1.235521	0.024821
C	0.026084	2.602139	0.012252
C	1.249340	3.403646	-0.280390
C	2.052776	3.117116	-1.397934
H	1.784499	2.284055	-2.040442
C	3.182644	3.882655	-1.682471
H	3.787562	3.646259	-2.553974
C	3.533190	4.953978	-0.855388
H	4.414676	5.549911	-1.075754
C	2.736577	5.258619	0.251856
H	2.998199	6.091640	0.899059
C	1.601009	4.497231	0.529717
H	0.980001	4.743223	1.386680
C	-1.202577	3.400995	0.289387
C	-2.009387	3.127376	1.407841
H	-1.738120	2.307813	2.066235
C	-3.145845	3.889641	1.674936
H	-3.753093	3.662940	2.547408
C	-3.499296	4.945695	0.829863
H	-4.385499	5.539430	1.036836
C	-2.698877	5.238431	-0.277999
H	-2.962506	6.059752	-0.939205
C	-1.557376	4.480141	-0.538810
H	-0.934127	4.716645	-1.396784

C	-1.229763	0.440210	0.025277
C	-1.407516	-0.634139	0.915473
H	-0.625058	-0.864851	1.632756
C	-2.574237	-1.386935	0.917829
H	-2.696987	-2.174276	1.655514
C	-3.604643	-1.125027	-0.005848
C	-3.423606	-0.069383	-0.915011
H	-4.173211	0.153011	-1.665937
C	-2.261087	0.699625	-0.887100
H	-2.145778	1.510923	-1.599209
N	-4.786441	-1.910546	0.031843
C	-5.917992	-1.465603	-0.772626
H	-5.762457	-1.638571	-1.852378
H	-6.071693	-0.393338	-0.629503
C	-7.198424	-2.158061	-0.318793
H	-7.430160	-1.917192	0.720543
H	-8.040879	-1.884155	-0.955818
N	-7.049511	-3.661432	-0.385266
C	-8.300950	-4.373809	0.037312
H	-8.145122	-5.446596	-0.078271
H	-9.121034	-4.038611	-0.598105
H	-8.501252	-4.127640	1.080233
C	-5.841823	-4.097803	0.413283
H	-5.736720	-5.176683	0.289093
H	-6.059096	-3.865451	1.457831
C	-4.602114	-3.361073	-0.072973
H	-3.766341	-3.684184	0.547487
H	-4.371707	-3.660302	-1.111032
H	-6.873001	-3.910146	-1.365899
C	1.422017	-0.691176	-0.797784
C	2.582555	-1.462552	-0.799218
H	2.628522	-2.325726	-1.452737
H	0.603817	-0.971663	-1.455622
H	2.280345	1.592527	1.548006
H	4.328878	0.262308	1.554294
C	5.043420	-2.903722	-0.980254
C	6.508589	-3.328583	-1.064627
H	6.601642	-4.135124	-1.800939
H	7.111178	-2.472267	-1.428364
H	4.743139	-2.498808	-1.950835
H	4.417887	-3.789756	-0.771343
H	4.789483	-3.164882	1.720913
H	5.315403	-1.508729	2.071741
H	7.468135	-1.865818	0.973150
H	7.187892	-3.125943	2.197975

(3) S_1 geometry of PIP-TPEH$^+_{(soln)}$:

H	9.114386	-4.463417	-0.073077
C	9.030758	-3.415189	0.233603
H	9.114386	-4.463417	-0.073077
H	9.639233	-3.269667	1.132707
H	9.453397	-2.786040	-0.573175
N	7.637715	-3.099628	0.528635
C	7.483700	-1.721453	0.989539
C	6.033477	-1.447648	1.374379
N	5.125890	-1.724884	0.251735
C	3.873108	-1.126990	0.215000
C	3.699270	0.229339	0.602308
C	2.471022	0.849055	0.523085
C	1.294696	0.168521	0.085319
C	0.037681	0.858046	0.008560
C	0.041001	2.342353	-0.029324
C	0.674387	2.992084	-1.150966
C	0.898003	2.278151	-2.365940
H	0.537084	1.257399	-2.444702
C	1.537687	2.865443	-3.449620
H	1.672824	2.292489	-4.363925
C	2.009430	4.182397	-3.373894
H	2.516916	4.637048	-4.219989
C	1.832800	4.897977	-2.181038
H	2.220532	5.910185	-2.094043
C	1.184900	4.323708	-1.095890
H	1.107382	4.883532	-0.170159
C	-0.611182	3.047167	1.051825
C	-0.774304	2.423425	2.322668
H	-0.360383	1.430596	2.472565
C	-1.424155	3.062128	3.370161
H	-1.511996	2.559083	4.330197
C	-1.966464	4.343513	3.199672
H	-2.481972	4.838302	4.017959
C	-1.849884	4.967617	1.950972
H	-2.292238	5.948109	1.791317
C	-1.191494	4.339949	0.900505
H	-1.160429	4.825417	-0.069122
C	-1.243532	0.189538	-0.034783
C	-1.484038	-1.088651	0.547168
H	-0.683363	-1.590416	1.079829
C	-2.734744	-1.677636	0.526426
H	-2.879326	-2.627273	1.033434
C	-3.835264	-1.042828	-0.090006

C	-3.624934	0.233900	-0.647625
H	-4.433778	0.762968	-1.139421
C	-2.375568	0.834353	-0.600324
H	-2.248333	1.816883	-1.043915
N	-5.099204	-1.676645	-0.077558
C	-6.260573	-0.891646	-0.476792
H	-6.303432	-0.729949	-1.568161
H	-6.222373	0.089991	0.001225
C	-7.544803	-1.562469	-0.001570
H	-7.568888	-1.640107	1.086973
H	-8.423894	-1.018321	-0.350101
N	-7.642455	-2.974026	-0.534087
C	-8.904132	-3.660967	-0.098799
H	-8.934976	-4.648616	-0.558780
H	-9.755509	-3.063312	-0.424473
H	-8.892572	-3.748230	0.987819
C	-6.406984	-3.759068	-0.152791
H	-6.491525	-4.746589	-0.609729
H	-6.422210	-3.852727	0.934888
C	-5.156079	-3.034368	-0.627925
H	-4.295320	-3.614776	-0.294982
H	-5.128402	-3.014708	-1.731620
H	-7.664797	-2.914799	-1.559259
C	1.488780	-1.182224	-0.324358
C	2.722549	-1.804082	-0.265276
H	2.790046	-2.836007	-0.588308
H	0.653753	-1.740601	-0.732581
H	2.388095	1.891320	0.814710
H	4.552106	0.811698	0.933166
C	5.310548	-3.070231	-0.293006
C	6.780412	-3.318062	-0.633693
H	6.889841	-4.355279	-0.969948
H	7.068348	-2.660812	-1.478484
H	4.728732	-3.170333	-1.211539
H	4.960493	-3.833207	0.421910
H	5.757968	-2.085898	2.230143
H	5.938160	-0.409383	1.689588
H	7.798775	-0.994068	0.214907
H	8.117577	-1.565113	1.869661

(4) S_0 geometry of PIP-TPE$_{(bulk)}$:

C	2.255700	-4.413300	11.498100

C	3.597200	-4.683500	11.414200
C	1.751200	-3.074300	11.916000
C	2.340800	-2.373600	12.983800
C	1.955600	-1.092000	13.336200
C	0.934800	-0.421700	12.627100
C	0.272600	-1.157500	11.626800
C	0.675500	-2.444400	11.273300
C	1.223900	-5.429500	11.146800
C	0.092800	-5.660700	11.950800
C	-0.865900	-6.610700	11.619600
C	-0.758100	-7.378000	10.436100
C	0.349400	-7.110000	9.602400
C	1.311500	-6.173300	9.961400
C	-0.361900	4.961000	13.799800
C	0.630400	1.381100	14.264100
C	0.770400	2.894600	14.352000
C	-0.273400	3.125400	12.226200
C	-0.373900	1.604700	12.080800
C	-4.760100	-11.020100	8.746400
C	-1.562700	-9.042400	8.824300
C	-2.454300	-10.277000	8.743600
C	-3.933700	-9.420100	10.356900
C	-3.088600	-8.161500	10.528800
C	4.652900	-3.626400	11.486600
C	4.610600	-2.486100	10.666600
C	5.584200	-1.494100	10.767000
C	6.618200	-1.620200	11.695700
C	6.701300	-2.766200	12.486300
C	5.738500	-3.767200	12.370900
C	4.148300	-6.065200	11.270600
C	3.816000	-7.084000	12.179500
C	4.406700	-8.342800	12.084700
C	5.324700	-8.614400	11.068700
C	5.675600	-7.610500	10.166700
C	5.103500	-6.345200	10.276800
N	0.641600	0.926800	12.877200
N	-0.338200	3.517400	13.632900
N	-1.697100	-8.371900	10.115900
N	-3.840600	-9.917600	8.993300
H	3.141300	-2.844400	13.535900
H	2.483300	-0.604600	14.147400
H	-0.569900	-0.709300	11.122600
H	0.156300	-2.958200	10.469500
H	-0.037400	-5.093000	12.866500
H	-1.692800	-6.761600	12.303400
H	0.467700	-7.617900	8.655400

H	2.145400	-5.997900	9.290300
H	-1.237400	5.376900	13.291900
H	-0.434500	5.208700	14.864200
H	0.544400	5.454200	13.398900
H	-0.324000	1.086800	14.724300
H	1.435600	0.902000	14.812600
H	1.755500	3.197000	13.948400
H	0.741100	3.204100	15.402300
H	-1.111900	3.584300	11.689200
H	0.663800	3.472400	11.750800
H	-0.242100	1.347400	11.027300
H	-1.385400	1.294600	12.389400
H	-5.784300	-10.639500	8.796400
H	-4.600900	-11.408300	7.736400
H	-4.651900	-11.855300	9.460900
H	-1.809200	-8.361100	7.990000
H	-0.525600	-9.358400	8.698700
H	-2.089200	-11.046600	9.447900
H	-2.394000	-10.695800	7.740200
H	-4.975800	-9.165100	10.571600
H	-3.613400	-10.191100	11.082000
H	-3.113900	-7.866700	11.576900
H	-3.550500	-7.350200	9.945200
H	3.793800	-2.376300	9.960400
H	5.529000	-0.614800	10.133000
H	7.357900	-0.837400	11.805800
H	7.512400	-2.862900	13.201800
H	5.807000	-4.658300	12.988800
H	3.102600	-6.875000	12.970300
H	4.176300	-9.104800	12.823600
H	5.794600	-9.590700	11.001200
H	6.394700	-7.819200	9.382300
H	5.389400	-5.558400	9.584900

(5) S_1 of PIP-TPE$_{(bulk)}$.

a. PL calculation:

Excited State 1: Singlet 2.6320 eV 471.07 nm f=0.4194 <S**2>=0.000

142 ->143 -0.70627

b. Optimized geometry:

C	2.246700	-4.406100	11.486300
C	3.659300	-4.675500	11.368000
C	1.760300	-3.111500	11.952900
C	2.434800	-2.343900	12.943100
C	2.044100	-1.064400	13.286400
C	0.956400	-0.430600	12.643600
C	0.241200	-1.200100	11.700000
C	0.627400	-2.488700	11.365400
C	1.248800	-5.406000	11.110700
C	0.036600	-5.551100	11.839400
C	-0.923800	-6.488500	11.509400
C	-0.755100	-7.355200	10.394200
C	0.438600	-7.202100	9.645600
C	1.408500	-6.284600	10.007800
C	-0.350400	4.952400	13.797000
C	0.656100	1.377700	14.269100
C	0.790300	2.891800	14.354000
C	-0.268700	3.109900	12.231100
C	-0.366600	1.587800	12.094400
C	-4.770000	-11.003200	8.748100
C	-1.558200	-9.051200	8.805400
C	-2.457100	-10.281400	8.749800
C	-3.924300	-9.413400	10.358400
C	-3.083900	-8.151100	10.531400
C	4.652600	-3.600000	11.346100
C	4.488100	-2.395100	10.619000
C	5.427100	-1.373900	10.704200
C	6.520100	-1.487600	11.567800
C	6.717000	-2.673600	12.284800
C	5.816100	-3.719900	12.156700
C	4.208700	-6.022300	11.307300
C	3.731100	-7.114900	12.080600
C	4.325000	-8.368300	11.973100
C	5.343600	-8.608200	11.046400
C	5.807700	-7.553300	10.247100
C	5.269500	-6.286800	10.391400
N	0.660100	0.920700	12.883500
N	-0.323900	3.508100	13.636700
N	-1.703500	-8.322300	10.066900
N	-3.839800	-9.908000	8.994300
H	3.285000	-2.782700	13.445200
H	2.622300	-0.535400	14.036200
H	-0.622100	-0.765400	11.216700
H	0.076500	-3.017700	10.594500
H	-0.129700	-4.920700	12.706400
H	-1.799700	-6.563600	12.141200

H	0.609800	-7.791200	8.757000
H	2.297900	-6.191400	9.397100
H	-1.229300	5.364200	13.291400
H	-0.418400	5.204800	14.860700
H	0.552600	5.446500	13.389400
H	-0.296100	1.080400	14.733000
H	1.463800	0.895100	14.811700
H	1.772600	3.198200	13.947000
H	0.761600	3.203600	15.403700
H	-1.112800	3.564700	11.699100
H	0.663700	3.456900	11.746600
H	-0.246400	1.321200	11.041500
H	-1.374500	1.278100	12.417000
H	-5.790300	-10.613000	8.797800
H	-4.613800	-11.393300	7.738500
H	-4.668100	-11.838100	9.463600
H	-1.786900	-8.392400	7.950500
H	-0.523000	-9.378200	8.704900
H	-2.097400	-11.042100	9.466300
H	-2.396700	-10.717000	7.753700
H	-4.964100	-9.158000	10.582300
H	-3.599700	-10.186000	11.079900
H	-3.076300	-7.885600	11.588100
H	-3.570300	-7.328200	9.987300
H	3.624900	-2.291600	9.970800
H	5.302400	-0.473800	10.110100
H	7.209100	-0.663400	11.695800
H	7.565500	-2.758800	12.958300
H	5.956300	-4.630200	12.731500
H	2.950300	-6.941200	12.814100
H	4.025900	-9.164800	12.646400
H	5.796900	-9.590900	10.968700
H	6.585400	-7.730500	9.511800
H	5.627800	-5.473700	9.768300

References:

[1] A. D. Becke, *J. Chem. Phys*., 1993, **98**, 5648-5652.
[2] C. Lee, W. Yang and R.G. Parr, *Phys. Rev. B*, 1988, **37**, 785-789.
[3] S. H. Vosko, L. Wilk and M. Nusair, *Can. J. Phys*., 1980, **58**, 1200-1211.
[4] P. J. Stephens, F. J. Devlin, C. F. Chabalowski and M. J. Frisch, *J. Phys. Chem*., 1994, **98**, 11623-11627.
[5] M. J. Frisch, G. W. Trucks, H. B. Schlegel, G. E. Scuseria, M. A. Robb, J. R. Cheeseman, G. Scalmani, V. Barone, B. Mennucci, G. A. Petersson, H. Nakatsuji, M. Caricato, X. Li, H. P. Hratchian, A. F. Izmaylov, J. Bloino, G. Zheng, J. L. Sonnenberg, M. Hada, M. Ehara, K. Toyota, R. Fukuda, J. Hasegawa, M. Ishida, T. Nakajima, Y. Honda, O. Kitao, H. Nakai, T. Vreven, J. A.

Montgomery, Jr., J. E. Peralta, F. Ogliaro, M. Bearpark, J. J. Heyd, E. Brothers, K. N. Kudin, V. N. Staroverov, T. Keith, R. Kobayashi, J. Normand, K. Raghavachari, A. Rendell, J. C. Burant, S. S. Iyengar, J. Tomasi, M. Cossi, N. Rega, J. M. Millam, M. Klene, J. E. Knox, J. B. Cross, V. Bakken, C. Adamo, J. Jaramillo, R. Gomperts, R. E. Stratmann, O. Yazyev, A. J. Austin, R. Cammi, C. Pomelli, J. W. Ochterski, R. L. Martin, K. Morokuma, V. G. Zakrzewski, G. A. Voth, P. Salvador, J. J. Dannenberg, S. Dapprich, A. D. Daniels, O. Farkas, J. B. Foresman, J. V. Ortiz, J. Cioslowski and D. J. Fox. *EM64L-Gaussian 09*, *Revision D.01*. Gaussian, Inc., Wallingford CT, 2013.

[6] J. Tomasi, B. Mennucci and R. Cammi, Quantum Mechanical Continuum Solvation Models. *Chem. Rev*., 2005, **105**, 2999-3094.

[7] S. Grimme, *J Comput. Chem.*, 2004, **25**, 1463-1473.

[8] F. Maseras and K. Morokuma, *J. Comput. Chem*., 1995, **16**, 1170-1179.

[9] M. Svensson, S. Humbel, R. D. J. Froese, T. Matsubara, S. Sieber and K. Morokuma, *J. Phys. Chem.*, 1996, **100**, 19357-19363.

[10] S. Dapprich, I. Komáromi, K. S. Byun, K. Morokuma and M. J. Frisch, *J. Mol. Struct. (THEOCHEM),* 1999, **461**, 1-23.

[11] T. Vreven, K. S. Byun, I. Komáromi, S. Dapprich, J. A. Jr. Montgomery, K. Morokuma and M. J. Frisch, *J. Chem. Theory Comput*., 2006, **2**, 815-826.

[12] A. D. Becke, *J. Chem. Phys*., 1993, **98**, 5648-5652.

[13] A. K. Rappe, C. J. Casewit, K. S. Colwell, W. A. GoddardIII and W. A. Skiff, *J. Am. Chem. Soc*., 1992, **114**, 10024-10035.

[14] D. Bakowies and W. Thiel, *J. Phys. Chem*., 1996, **100**, 10580-10594.

[15] Q. Y. Wu, C. M. Deng, Q. Peng, Y. L. Niu and Z. G. Shuai, Quantum Chemical Insights into the Aggregation Induced Emission Phenomena: A QM/MM Study for Pyrazine Derivatives. *J. Comput. Chem*., 2012, **33**, 1862-1869.

[16] Q. Y. Wu, T. Zhang, Q. Peng, D. Wang and Z. G. Shuai, Aggregation Induced Blue-Shifted Emission-the Molecular Picture from a QM/MM Study. *Phys. Chem. Chem. Phys*., 2014, **16**, 5545-5552.
